# Disrupting Pheromone Signaling in Insects: Design, Synthesis, and Evaluation of an Inhibitor

**DOI:** 10.1021/acsomega.5c06376

**Published:** 2026-01-16

**Authors:** Pratikshya Paudel, Ishani Ray, Omar Al Danoon, James R. Howard, Josef M. Maier, Sarah R. Moor, Anna M. Lidskog, Eric V. Anslyn, Smita Mohanty

**Affiliations:** † Department of Chemistry, 7618 Oklahoma State University, Stillwater, Oklahoma 74078, United States; ‡ Department of Chemistry, 12330 University of Texas at Austin, Austin, Texas 78712, United States

## Abstract

Sex pheromones mediate mate recognition in insects through interactions with pheromone-binding proteins (PBPs). Targeting this pathway with pheromone analogs offers a species-specific approach to pest management. Here, we report the design, synthesis, and evaluation of a pheromone analog, (6*E*,11*Z*)-heptadeca-6,11-dien-1-yl acetate as an inhibitor of *Antheraea polyphemus* PBP1 (ApolPBP1). Molecular docking predicted potential binding orientations and interactions within the pocket. Fluorescence-based binding assays revealed a 4-fold higher dissociation constant, and 2D [^1^H, ^15^N] HSQC NMR titrations confirmed reduced affinity, with ApolPBP1 transitioning to the bound state only at higher analog concentrations. Overall, these findings highlight the sensitivity of PBPs to subtle structural modifications in their ligands and emphasize the importance of preserving key molecular interactions for effective binding.

## Graphical abstract

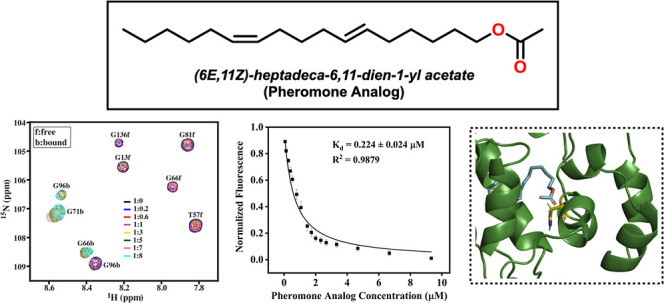

## Introduction

Olfaction is vital for animal survival, guiding critical behaviors such as foraging, mating, and predator avoidance.^1^ This sensory modality relies on semiochemicals- small, volatile, organic compounds that mediate communication by triggering and modulating behaviors.^2^ Among these, pheromones represent a specialized subclass released by animals to facilitate intraspecific communication.^2^ These molecules convey crucial information about an individual’s behavioral state, physiological condition, and reproductive status.^2^ Pheromones regulate social interactions including mate attraction, territorial marking, trail following, alarm signaling, and other biologically significant responses.^2,3^

In lepidopteran moths, female-emitted sex pheromones play a pivotal role in eliciting mating behaviors in conspecific males.^4^ Detection of these chemical cues is facilitated by pheromone-binding proteins (PBPs), which are abundantly expressed in the male antennae.^5–7^ PBPs selectively bind hydrophobic pheromone molecules and transport them across the aqueous sensillum lymph to specific olfactory receptor neurons (ORNs).^5,6^ This ligand–protein interaction initiates a receptor-mediated signal transduction cascade, ultimately guiding males toward receptive females and triggering courtship behavior.^4,8^

PBPs are small, water-soluble proteins with a molecular mass typically ranging from 14 to 16 kDa.^9^ They exhibit a highly helical structure, generally consisting of six to seven α-helices that fold into a compact architecture enclosing a hydrophobic binding pocket, the primary site for ligand binding.^10–12^ The protein’s structural stability is conferred by three disulfide bridges formed by six conserved cysteine residues, which are critical for maintaining the helical framework and overall protein conformation.^13–15^

The giant silk moth *Antheraea polyphemus* (Apol), a member of the Saturniidae family, is one of the largest silk moths in the world and has long served as a key model organism for investigating insect olfaction. It was the first species in which a pheromone-binding protein (ApolPBP1) was identified, making it a cornerstone for understanding lepidopteran chemical communication.^16^ Since its discovery in 1981, ApolPBP1 and related PBPs have been cloned, expressed, and structurally characterized, enabling detailed investigations into their ligand-binding properties and conformational dynamics.^12,17–32^

PBPs facilitate pheromone transport by undergoing a pH-dependent conformational switch that regulates ligand binding and release.^12,18,20,21,23,26,27,29–33^ At physiological pH within the sensillum lymph, these proteins bind ligands within their hydrophobic pockets. When the PBP–ligand complex approaches the more acidic environment near the ORNs, the protein undergoes a structural transition that induces ligand dissociation. Specifically, at high pH, the protein adopts the ligand-bound “open” or PBP^B^ conformation, characterized by an unstructured solvent-exposed C-terminal region.^12,21,22,26^ In contrast, at low pH, the C-terminus folds into an α-helix and inserts into the hydrophobic pocket, displacing the ligand to form the ligand-free “closed” or PBP^A^ conformation.^12,21,22,26^ This pH-sensitive switching mechanism ensures efficient directional transport and timely release of pheromones, critical steps in the olfactory signal transduction pathway. Notably, in the absence of a ligand, the C-terminus occupies the pocket, irrespective of pH.

Moth sex pheromones are fatty acid-derived hydrophobic compounds that play a central role in species-specific communication.^2^ In *A. polyphemus*, the primary sex pheromone is (6*E*,11*Z*)-hexadeca-6,11-dien-1-yl acetate (Figure 1). Upon delivery to the ORNs, interaction with specific receptor proteins initiates a signal transduction cascade that leads to sensory neuron depolarization and ultimately triggers behavioral responses in male moths. This olfactory signaling event enables males to locate conspecific females and initiate mating. As key mediators of pheromone detection, PBPs are essential for reproductive success and the maintenance of population dynamics.^34,35^ Their ligand specificity and central role in chemical communication make them attractive molecular targets for the development of species-specific, environmentally benign semiochemicals designed to disrupt mating behavior via competitive inhibition.

**Figure 1:**
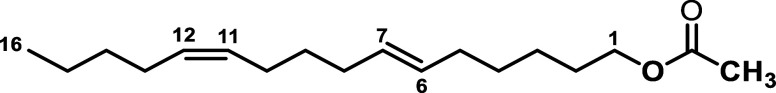
Chemical structure of the sex pheromone of *Antheraea polyphemus*, (6*E*,11*Z*)-hexadeca-6,11-dien-1-yl acetate.

Targeting male moth responses to female-emitted pheromones through competitive inhibition and sensory interference offers a species-specific and environmentally sustainable alternative to conventional chemical pesticides. Unlike broad-spectrum insecticides, which often promote resistance and cause ecological harm by affecting nontarget species, pheromone-based strategies minimize environmental impact while preserving natural ecosystems. This highlights the need for systematic, structure-based rational design, synthesis, and validation of effective pheromone inhibitors. Although *A. polyphemus* is not an agricultural pest, ApolPBP1, the most extensively characterized lepidopteran PBP, serves as a valuable model for developing and evaluating selective inhibitors. Its well-defined structure and binding properties provide a strong foundation for investigating structure–function relationships and guiding the rational design of competitive inhibitors for integrated pest management.

Here, we report the rational design, synthesis, and biochemical evaluation of a pheromone analog, (6*E*,11*Z*)-heptadeca-6,11-dien-1-yl acetate, that targets ApolPBP1. Employing a structure-based inhibitor design supported by computational modeling and experimental validation, we investigated the effects of a subtle structural modification by extending the aliphatic chain of the natural pheromone. We hypothesized that this modification would enhance hydrophobic interactions within the protein’s binding pocket, potentially increasing the binding energy and, consequently, improving the binding affinity.

The analog’s affinity with ApolPBP1 was measured using fluorescence-based competitive binding assays, complemented by high-resolution NMR titration experiments performed at pH 6.5 to characterize its binding. Unexpectedly, the results revealed that while the analog is capable of binding ApolPBP1, its affinity is approximately 4-fold lower than that of the native ligand. These findings underscore the sensitivity of ligand–PBP interactions to even minimal structural modifications and emphasize the importance of maintaining key molecular contacts for optimal binding.

## Methods

### Expression and Purification of Protein, ApolPBP1

The recombinant unlabeled and ^15^N-labeled recombinant ApolPBP1 proteins were expressed in *E. coli* Origami cells using a pET21a vector.^26^ A saturated overnight bacterial culture in Lysogeny broth (LB) containing ampicillin, tetracycline, and kanamycin was diluted (1:50 v/v) in LB or M9 minimal medium and grown at 37 °C until an A_600_ of 0.5–0.6 was reached. Expression was induced with addition of 1 mM isopropyl β-D-1-thiogalactopyranoside (IPTG), and cells were cultured for 4 h (LB) or 16 h (minimal media) at 30 °C. Bacterial cells were harvested by centrifugation (Sorvall LYNX 4000 centrifuge) at 8000 rpm and 4 °C for 30 min. The cell pellet was resuspended in the bacterial protein extraction reagent (B-PER, Thermo Scientific) and subjected to sonication. The cell lysate was centrifuged, and the recombinant protein was purified as previously reported.^24,26^

### Delipidation of ApolPBP1

The protein was buffer exchanged to 50 mM sodium citrate buffer pH 4.5 (buffer A) and concentrated to 1 mL using a Millipore ultrafiltration concentrator (capacity 15 mL, molecular-weight cut off (MWCO) of 3000). Delipidation was performed using two Lipidex-1000 columns (5 and 3 cm heights) equilibrated with Buffer A. The protein was incubated overnight with the first lipidex column while gentle shaking. Subsequently, the protein was collected and incubated again overnight in a second lipidex column with gentle shaking. The protein was eluted into a fresh Millipore concentrator of MWCO 3000, and the column was washed with Buffer A. The washes were combined with the eluted protein. Finally, the delipidated ApolPBP1 was concentrated and buffer exchanged to 20 mM phosphate buffer at pH 6.5 for fluorescence studies or 50 mM phosphate buffer, pH 6.5, 1 mM EDTA, 0.01% sodium azide, and 5% deuterated water for NMR studies.

### Design of the Pheromone Analog, (6*E*,11*Z*)-heptadeca-6,11-dien-1-yl Acetate

The design of the pheromone analog, (6*E*,11*Z*)-heptadeca-6,11-dien-1-yl acetate, was guided by the structural characteristics of the natural pheromone, (6*E*,11*Z*)-hexadeca-6,11-dien-1-yl acetate, which exhibits high binding affinity to ApolPBP1. The primary objective was to introduce a subtle structural modification aimed at increasing the hydrophobicity of the molecule while maintaining its overall functional characteristics. The aliphatic hydrocarbon chain was extended by a single methylene unit, increasing its length from 16 to 17 carbons to explore the effect of increased hydrophobic interactions within the protein’s binding pocket. The acetate group was retained to preserve the terminal functionality essential for residue-specific interactions within the binding pocket, while the double bonds at the sixth and 11th positions were conserved to maintain a close structural resemblance to the native ligand and minimize perturbations to its overall geometry.

### Synthesis and Characterization of the Pheromone Analog, (6*E*,11*Z*)-heptadeca-6,11-dien-1-yl Acetate

The synthesis of the pheromone analog (**12** in Scheme 1) was adapted from previously reported synthetic protocols by Millar^36^ and Bestmann.^37^ The route utilizes two different alkynes (**1** and **7**) that can be independently functionalized and subsequently ligated via an S_N_2 reaction between alkyne **8** and bromide **6**. Bromide **6** was synthesized through initial THP protection on pentyne alcohol **1**, followed by alkylation with pentyl iodide. After deprotection of the THP group, the resulting alkynol **4** was stereoselectively reduced to (4*Z*)-dec-4-en-1-ol **5** with an in situ generated P-2 nickel catalyst and hydrogen. Conversion into bromide **6** was achieved through treatment with methanesulphonyl chloride and triethyl amine, followed by LiBr in acetone.^36^ To complete the carbon backbone, THP-protected heptyne alcohol **8** was coupled to bromide **6** through an S_N_2 reaction followed by THP deprotection. Finally, dissolving metal reduction to selectively reduce the alkyne to the *trans* isomer, followed by acetylation of the terminal alcohol yielded pheromone mimetic **12**.^37^ Characterization using ^1^H and ^13^C NMR spectroscopy and ESI-MS confirmed the identity and purity of the final product (Figures S1–S15).

**Scheme 1:**
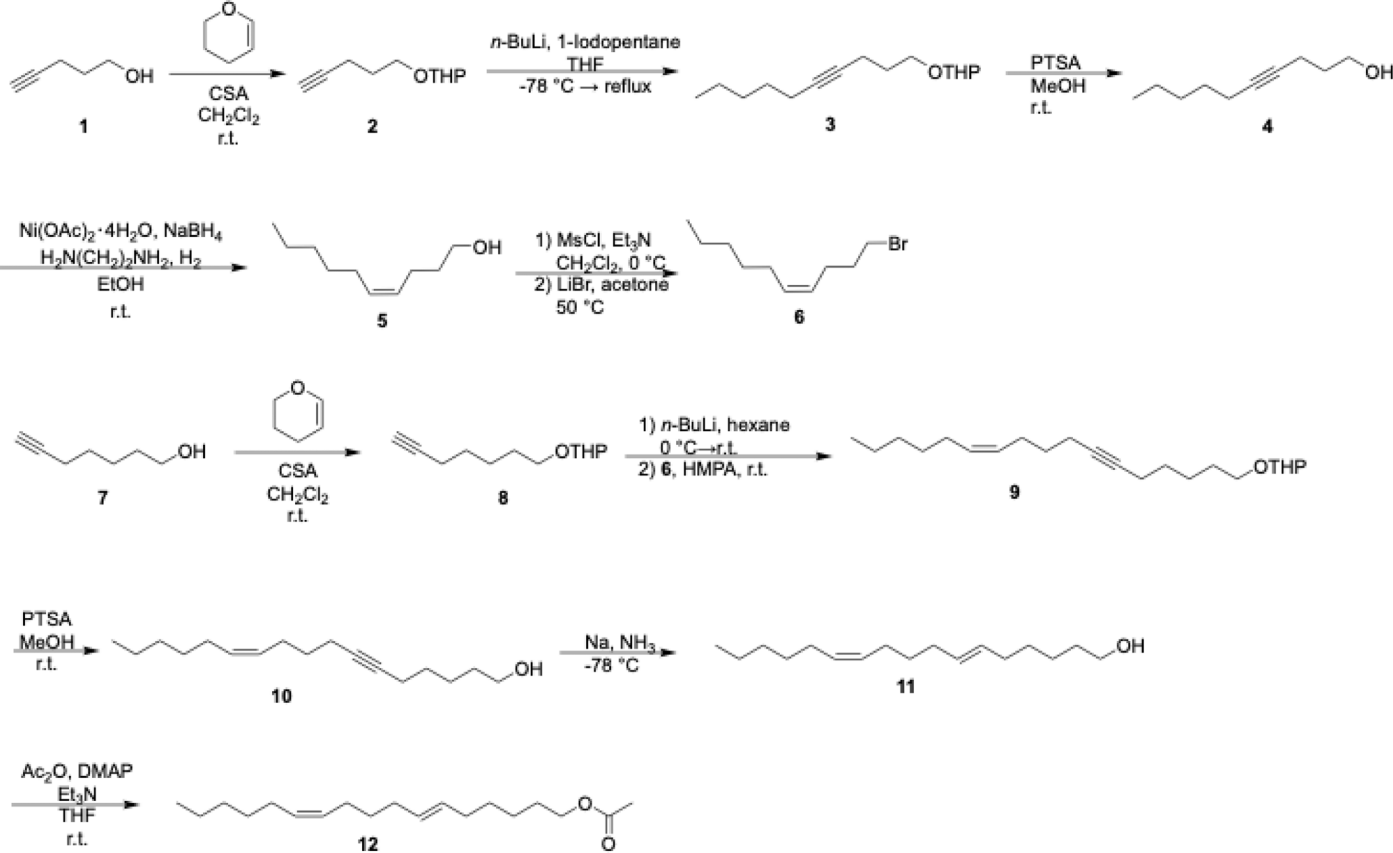
Synthetic Pathway to Pheromone Analog **12**. CSA = Camphor Sulfonic Acid, PTSA = *p*-Toluene Sulfonic Acid, MsCl = Methanesulfonyl Chloride, HMPA = Hexamethylphosphoramide, DMAP = 4-Dimethylaminopyridine

### Fluorescence Spectroscopy

The fluorescence binding assays were performed at room temperature using a PerkinElmer LS-55 fluorescence spectrometer housed in the Mohanty Laboratory, Department of Chemistry, Oklahoma State University. The experimental procedure followed previously published protocols for OnubPBP3 ^38^ with parameters optimized for ApolPBP1. All binding assays were carried out in triplicate sets.

For direct binding assays, 1 μM ApolPBP1 was titrated with increasing concentrations of 1-*N*-phenylnaphthylamine (NPN; 0–25.6 μM) from a 2 mM stock solution in methanol. Following each addition, samples were allowed to equilibrate for 2 min prior to the spectral acquisition. Background fluorescence from NPN in buffer was subtracted, and the binding constant (*K*_NPN_) was determined by fitting data to a single-site binding model using Origin 2019 software:

y=Bx/(k+x)

where *B* represents the maximum fluorescence intensity (the maximum binding capacity), *k* is the dissociation constant, *x* is the NPN concentration, and *y* is the fluorescence intensity at the specific ligand concentration.

For competitive binding experiments, a 1:1 ApolPBP1–NPN complex was titrated with increasing concentrations (0–11 μM) of either the natural pheromone or the synthetic analog. Fluorescence emission at 409 nm was recorded following approximately 10 min of incubation after each addition. Control experiments were performed by titrating the analog into NPN in buffer without protein to account for any nonspecific effects. The half-maximal inhibitory concentration (IC_50_) value of the natural and synthetic analog was determined by fitting the fluorescence data using the equation below:

y=1−x/(k+x)

where *x* is the analog concentration, *y* is the fluorescence intensity at specific ligand concentration, and *k* is the IC_50_. Data analysis was performed using Origin 2019 software.^39^

The dissociation constants (*K*_d_) were calculated using

$$K_{d}={[IC}_{50}]/(1+\left[NPN\right]/\left(K_{NPN}\right)$$

where [NPN] is the free NPN concentration and *K*_NPN_ is the dissociation constant for the NPN–ApolPBP1 complex. The IC_50_ and *K*_d_ values were reported with precision obtained from the curve fitting.

### NMR Measurements

NMR experiments were performed at 35 °C on a Bruker Neo 800 MHz spectrometer equipped with a triple-resonance ^1^H/^13^C/^15^N TCI cryoprobe at the Oklahoma Statewide Shared (OSS) Nuclear Magnetic Resonance Facility, Oklahoma State University, Stillwater, Oklahoma. For ligand titration experiments, NMR samples contained 350 μL of 545 μM uniformly ^15^N-labeled ApolPBP1, prepared as described under “Expression and Purification” and “Delipidation”, and placed in a shaped NMR tube (Bruker BioSpin). The two-dimensional [^1^H,^15^N] heteronuclear single quantum coherence (HSQC) spectrum of free ApolPBP1 at pH 6.5 was acquired as a reference. Titrations were carried out by incremental addition of the synthesized ligand (6*E*,11*Z*)-heptadeca-6,11-dien-1-yl acetate, from a 50 mM stock solution to final concentrations ranging from 0 to 3.82 mM. HSQC spectra were recorded after each addition. All NMR data were processed using NMRPipe^40^ and analyzed with NMRFAM-SPARKY.^41^

### Molecular Docking

Molecular docking was performed to predict the binding interactions and free binding energy (ΔG_B_) of the analog, in order to evaluate its potential as a competitive inhibitor of the natural pheromone. To further elucidate the molecular basis of ligand recognition, we also conducted docking simulations with additional naturally occurring ligands from the same chemical family. These included (6*E*,11*Z*)-hexadeca-6,11-dien-1-ol, a known pheromone metabolite of the *A. polyphemus* moth, and bombykol, (10*E*,12*Z*)-hexadeca-10,12-dien-1-ol, the primary sex pheromone of the silk moth, *Bombyx mori*. The three-dimensional structure of ApolPBP1 was obtained from the RCSB Protein Data Bank (PDB ID: 1QWV).^26^ Both ligands were built and energy-minimized in Avogadro^42^ (version 1.2.0) using the Merck Molecular Force Field (MMFF94) and were saved in the Mol2 format for subsequent docking analysis.

Docking studies were performed using CB-Dock (version 2), a web-based blind docking tool that utilizes AutoDock vina to identify potential binding cavities in the protein and ranks docking poses based on Vina scores.^43,44^ The resulting docked protein–ligand complexes were visualized and analyzed using PyMol (version 2.6.2) and LigPlot+ (version 2.2.9) to investigate ligand–protein interactions, including hydrogen bonding and residue contacts within the binding pocket.

## Results

### Pheromone Analog Design and Synthesis

The pheromone analog, (6*E*,11*Z*)-heptadeca-6,11-dien-1-yl acetate, was synthesized by incorporating an additional methylene group into the structure of the natural pheromone, (6*E*,11*Z*)-hexadeca-6,11-dien-1-yl acetate, resulting in a 17-carbon analog. This structural modification was intended to increase the hydrophobicity of the molecule, with the aim of enhancing its binding affinity to ApolPBP1. Computational docking predicted a binding energy (ΔG_B_) of −5.4 kcal/mol for the analog, compared to −5.7 kcal/mol for the natural pheromone, suggesting that the analog may act as a potential competitive inhibitor. The chemical structure of the purified compound was confirmed by NMR spectroscopy and mass spectrometry (Figures S1–S15). The synthesis yielded 100 mg of the pure analog, sufficient for fluorescence-based binding assays and additional biophysical studies.

### Ligand Binding Affinity by Fluorescence

The extrinsic fluorescence probe 1-*N*-phenylnaphthylamine (NPN) was used to evaluate the binding affinity of ApolPBP1 to both the natural pheromone and the synthesized analog. In the absence of protein, NPN exhibits minimal fluorescence; however, upon addition of ApolPBP1, the fluorescence intensity increases significantly (Figure 2A), indicating a protein–probe complex. The dissociation constant (*K*_NPN_) for the ApolPBP1–NPN complex was determined to be 1.24 ± 0.06 μM (Figure 2B and Table 1).

**Figure 2:**
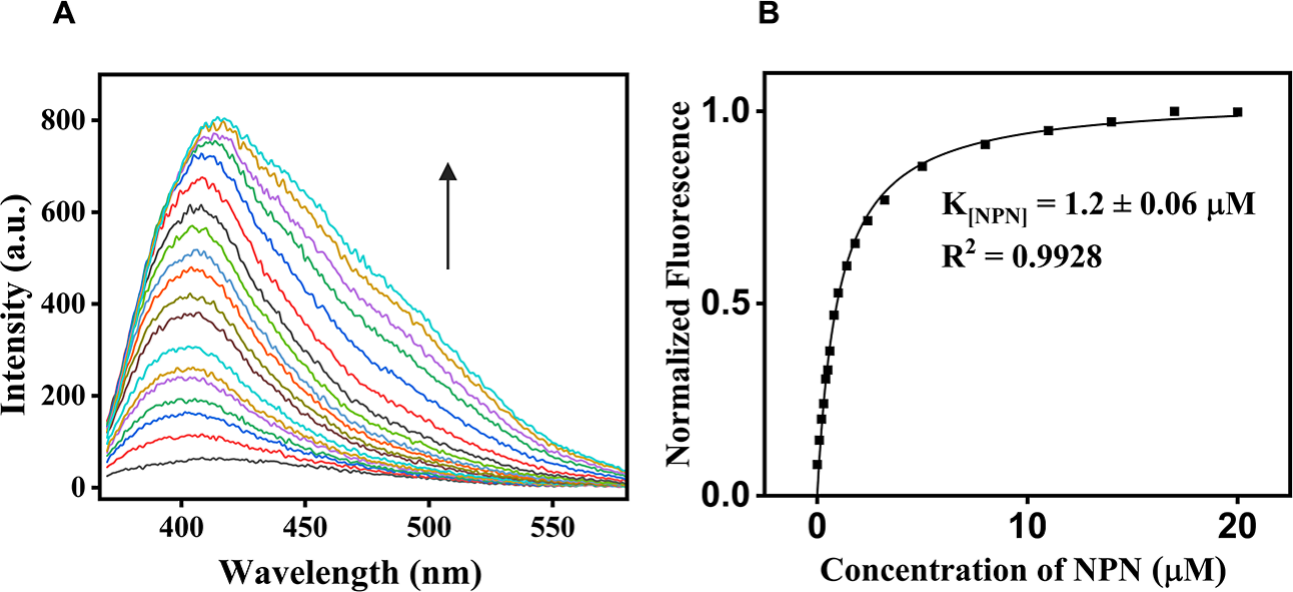
Binding of NPN to ApolPBP1 monitored by fluorescence spectroscopy: (A) fluorescence emission spectra of ApolPBP1–NPN complex upon titration with increasing concentrations of NPN. Emission spectra were recorded in the range of 370–600 nm with an excitation wavelength of 337 nm. (B) Plot of normalized fluorescence intensity as a function of NPN concentration.

**Table 1:** Dissociation Constants of NPN, Pheromone, and Pheromone Analog and IC_50_ Values of Pheromone and Analog to ApolPBP1

ligand classification	ligand name	IC_50_ (μM)	*K*_d_ (μM)
external probe	1-*N*-phenylnaphthylamine (NPN)	NA	1.24 ± 0.06
natural pheromone	(6*E*,11*Z*)-hexadeca-6,11-dien-1-yl acetate	0.105 ± 0.006	0.058 ± 0.004
pheromone analog	(6*E*,11*Z*)-heptadeca-6,11-dien-1-yl acetate	0.587 ± 0.046	0.224 ± 0.024

Competitive displacement binding assays were performed by titrating the natural pheromone (Figure S16A) or the analog (Figure 3A) into the ApolPBP1–NPN complex. These experiments yielded half-maximal inhibitory concentration (IC_50_) values of 0.105 μM for the pheromone and 0.587 μM for the analog. The dissociation constants *K*_d_ of the natural pheromone was calculated to be 0.058 ± 0.004 μM (Figure S16B), which is in close agreement with previously reported values obtained using AMA as the external probe.^26^ For analog, the *K*_d_ was determined to be 0.224 ± 0.024 μM (Figure 3B and Table 1), indicating a 4-fold lower binding affinity of the analog compared to the native ligand.

**Figure 3:**
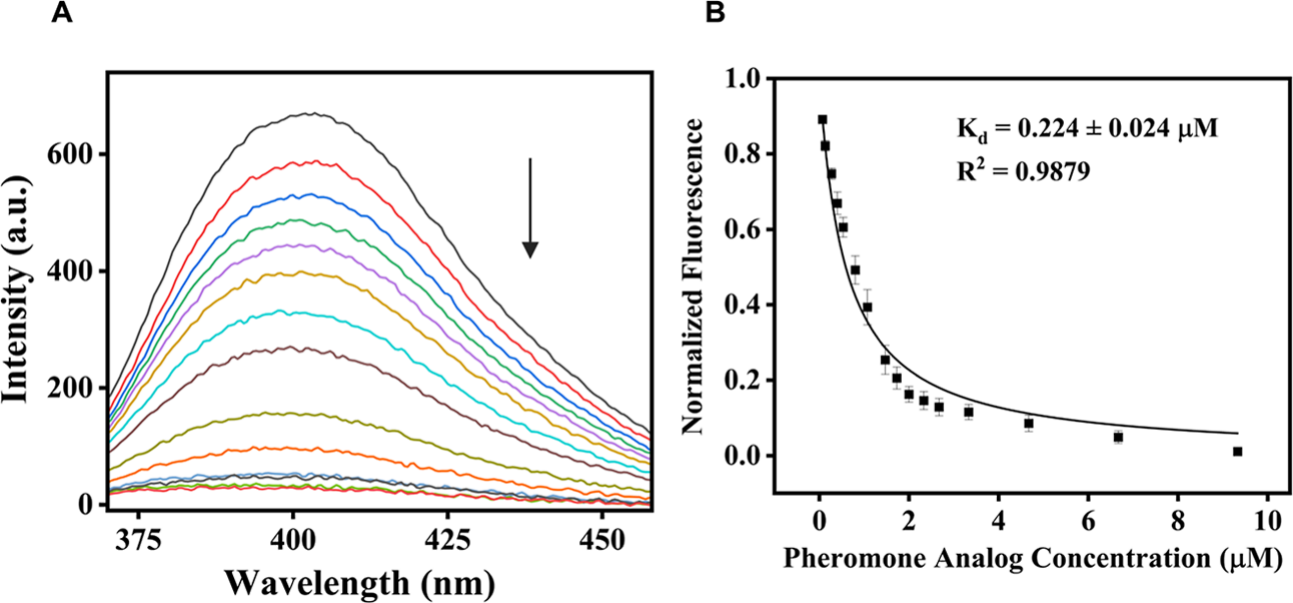
Competitive displacement of NPN from the ApolPBP1–NPN complex by the pheromone analog: (A) fluorescence emission spectra of the complex upon titration with increasing concentrations of the pheromone analog. (B) Plot of normalized fluorescence intensity.

### Effect of the Pheromone Analog on ApolPBP1 Conformation by NMR

ApolPBP1 undergoes a well-characterized conformational transition from a ligand-free “closed” state (PBP^A^) to a ligand-bound “open” state (PBP^B^) upon ligand binding at pH values above 6.0, as previously reported.^21,22,26^ This transition can be effectively monitored using 2D [^1^H, ^15^N] HSQC NMR spectroscopy.^26^ The 2D [^1^H, ^15^N] HSQC spectrum provides a high-resolution structural fingerprint of the protein, in which each nonproline residue typically gives rise to a distinct cross-peak corresponding to its amide proton and nitrogen chemical shifts. These chemical shifts are highly sensitive to the local environment and are influenced by factors such as ligand binding, pH changes, and temperature variations.^26^ Shifts in cross-peak positions (chemical shift perturbations) and/or changes in peak intensities are indicative of conformational changes. Thus, HSQC spectroscopy serves as a powerful tool for probing the structural dynamics of proteins and distinguishing between their ligand-free and ligand-bound states.

To investigate the effect of the pheromone analog on ligand-free ApolPBP1, a series of 2D HSQC NMR titration experiments were performed at pH 6.5. The protein was titrated with increasing concentration of the analog, with protein-to-ligand molar ratios ranging from 1:0 to 1:8 (Figure 4). At a 1:1 ratio, distinct changes in the resonance pattern were observed, including the appearance of cross-peaks corresponding to the ligand-bound conformation. Complete saturation was achieved at a 1:5 ratio, at which point the protein fully transitioned from the ligand-free state to the ligand-bound state (Figure 5C).

**Figure 4:**
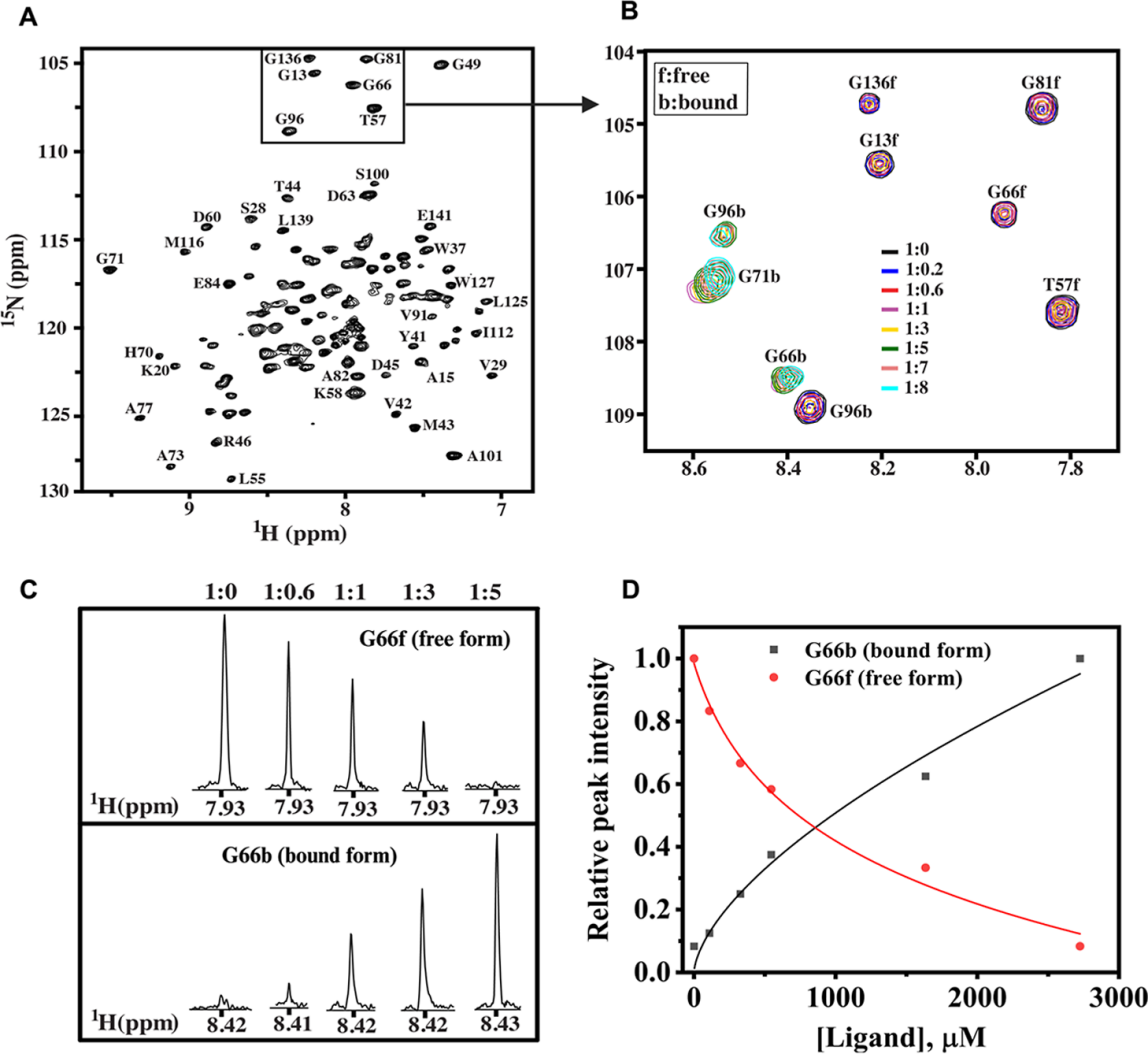
2D [^1^H, ^15^N] HSQC titration spectra of ApolPBP1 with the analog: (A) ligand-free ApolPBP1 at pH 6.5, with resonance assignments based on Mohanty et al.^45^ (B) Overlay of an expanded region (highlighted by rectangular insets in A) of the HSQC spectra recorded at increasing protein-to-ligand molar ratios, as indicated by color. (C) One-dimensional slices along the ^1^H axis taken at the center of Gly66 cross-peaks, showing the signal in both free and bound states across titration points. (D) Plot of ligand concentration against the ^1^H signal intensity of Gly66.

**Figure 5:**
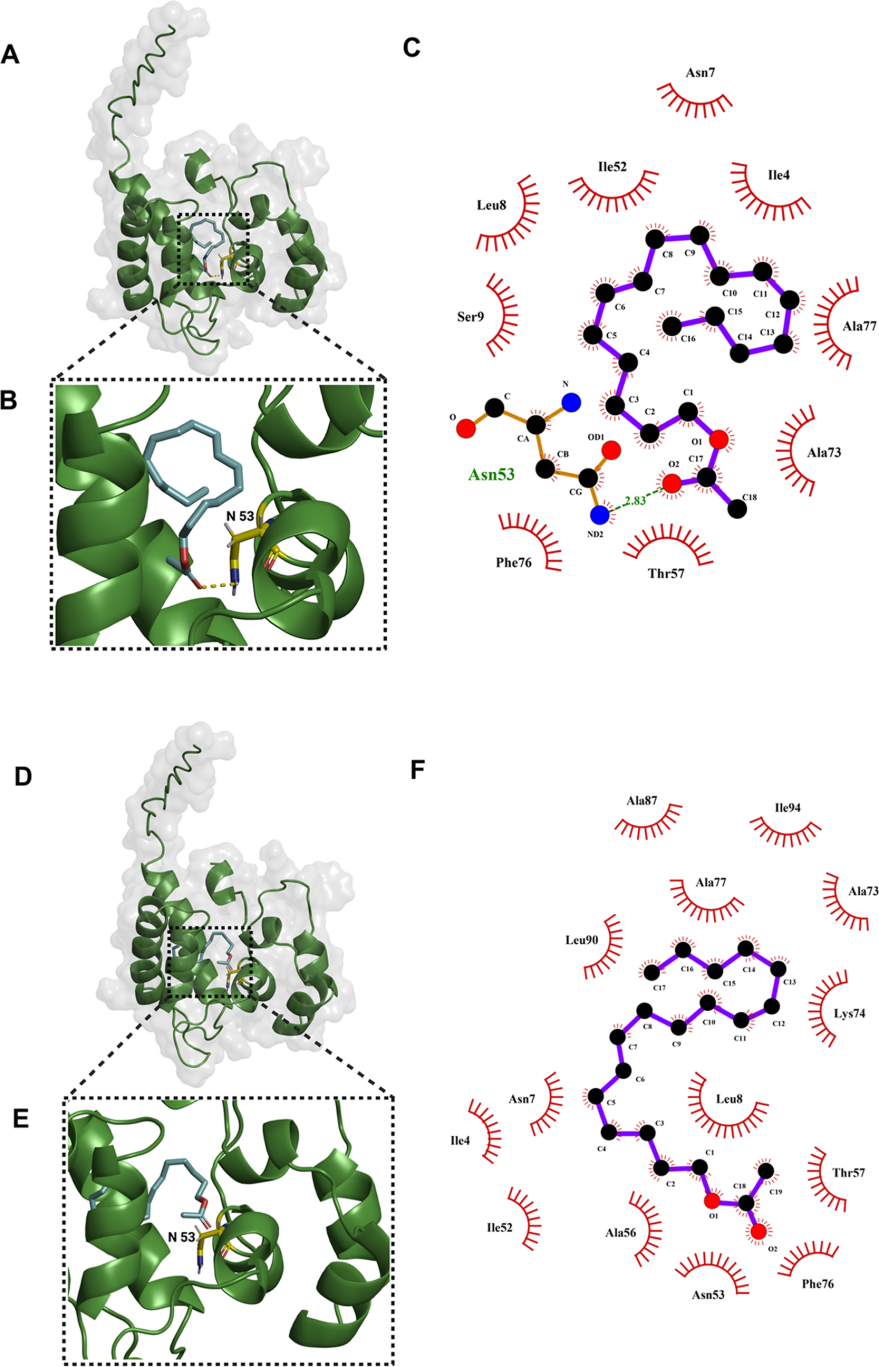
Molecular docking studies of the pheromone and the pheromone analog with ApolPBP1 (PDB ID: 1QWV). Both ligands are shown in *cyan*, with oxygen atoms highlighted in *red*. (A) Docked pose of the natural pheromone within the ApolPBP1 binding pocket. (B) Close-up view of the pheromone-binding site within ApolPBP1. (C) LigPlot representation showing key interactions. (D) Docking pose of the pheromone analog within the same binding pocket. (E) Close-up of the analog-binding site within ApolPBP1. (F) LigPlot diagram of interactions between ApolPBP1 and the pheromone analog.

### Molecular Docking Studies

Molecular docking analyses were performed using CB-Dock to predict the binding interactions and binding free energies (Δ*G*_B_) of ApolPBP1 with the natural pheromone and its analog. Among the five docking poses generated for each ligand, the pose featuring ligand binding within the largest predicted hydrophobic pocket, yielding binding energies of −5.7 kcal/mol for the pheromone and −5.4 kcal/mol for the analog, was used for comparison (Figure 5). Similarly, the docking was performed for two additional natural ligands: (6*E*,11*Z*)-hexadeca-6,11-dien-1-ol, a known metabolite of the *A. polyphemus* pheromone, and bombykol (10*E*,12Z)-hexadeca-10,12-dien-1-ol, the primary sex pheromone of *B. mori*, which showed binding energies of −4.9 and −5.3 kcal/mol, respectively (Figure S17). These selected poses were used to compare ligand conformations, energetic profiles, and key molecular interactions with those of the analog (Table S1).

The natural pheromone interacts primarily with residues Ile4, Asn7, Leu8, Ser9, Ile52, Asn53, Thr57, Ala73, Phe76, and Ala77, whereas the analog engages a broader set of residues, including Ile4, Asn7, Leu8, Ile52, Asn53, Ala56, Thr57, Ala73, Lys74, Phe76, Ala77, Ala87, Leu90, and Ile94 (Figure 5A–F). Comparable interactions with key binding site residues were also observed for the other ligands examined (Table S1). These interacting residues help define the conserved hydrophobic core of the binding pocket along with variable peripheral contacts that may contribute to differences in binding affinity and specificity. Notably, these interactions are consistent with the previously reported solution NMR structure of ApolPBP1.^25^

## Discussion

This study investigated the structure-based design, synthesis, and biophysical characterization of a pheromone analog, (6*E*,11*Z*)-heptadeca-6,11-dien-1-yl acetate, to evaluate its binding interactions with ApolPBP1 and its potential to act as a competitive inhibitor. The analog was rationally designed, by introducing an additional methylene group into the aliphatic chain of the natural pheromone, (6*E*,11*Z*)-hexadeca-6,11-dien-1-yl acetate, thereby extending the hydrophobic tail to potentially enhance interactions within the protein’s binding pocket. We hypothesized that this structural modification would increase the binding affinity and interfere with natural pheromone recognition.

To test this hypothesis, the analog was synthesized through a series of steps that preserved the core structure of the natural pheromone while extending its hydrophobic tail. The identity and purity of intermediates and the final product were confirmed by 1D and 2D ^1^H and ^13^C NMR (Figures S1–S15) and high-resolution mass spectrometry (HRMS), ensuring structural integrity for subsequent biophysical analyses.

Fluorescence-based binding assays (Figures 2 and 3) revealed an approximately 4-fold decrease in binding affinity for the analog compared to the natural pheromone, as indicated by higher IC_50_ and *K*_d_ values (Table 1; Figure S16). Likewise, 2D HSQC NMR titrations showed that full conversion to the bound state required a 5-fold molar excess of the analog (Figure 4), whereas the natural pheromone reached saturation at a 1:1 molar ratio, as reported previously.^26^ Collectively, these findings refute our initial hypothesis and demonstrate that chain elongation by a single carbon atom diminished the binding affinity rather than enhanced binding affinity. This outcome underscores the structural precision required for an optimal ApolPBP1–ligand interaction and the fine balance between increasing hydrophobicity and preservation of key polar contacts.

Consistent with our observations, similar trends have been reported in PBPs.^19,46–51^ Notably, studies on ApolPBPs have documented over a 1000-fold difference in binding affinity among closely related pheromone structures, highlighting the remarkable selectivity of these proteins.^46^ Because protein–ligand interaction is fundamentally governed by steric and physicochemical complementarity, effective binding requires that the binding pocket’s volume, shape, and chemical environment closely match those of the ligand.^47–50^ These reports collectively support that binding is highly sensitive to subtle structural features of the ligand, including chain length, double-bond position, and steric geometry that influence how well the ligand fits within the binding pocket.^19,51^

To rationalize the observed decrease in affinity, molecular docking was performed to further characterize the ligand–protein interactions and assess how structural variations affect the binding orientation and strength. Comparative analysis revealed that the natural pheromone, a 16-carbon acetate with (6*E*,11*Z*) double bonds, exhibited the strongest predicted binding affinity (−5.7 kcal/mol) (Figure 5A–C and Table S1), whereas the analog showed a slightly weaker predicted affinity (−5.4 kcal/mol, Figure 5D–F). The negative binding energies indicate a favorable complex formation. The modest difference in binding affinity suggests that the natural pheromone achieves optimal steric complementarity and favorable interactions within both the hydrophobic and polar regions of the binding pocket. In contrast, the additional methylene group in the analog likely introduces some steric strain, reducing geometric fit. This decrease in the induced fit may perturb packing, cause unfavorable positional displacement, and weaken key stabilizing contacts within the pocket.

To further probe how different chemical features, such as headgroup polarity and double-bond position, affect binding, additional docking simulations were performed with related compounds, including the natural pheromone metabolite and bombykol (Figure S17). In the *A. polyphemus* pheromone metabolite, the acetate headgroup is replaced by a hydroxyl group. This compound, (6*E*,11*Z*)-hexadecadienyl-1-ol, displayed a lower predicted affinity (−4.9 kcal/mol), consistent with the loss of polar interactions with residues such as threonine and asparagine that likely stabilize the ester moiety in the natural pheromone (Table S1). Bombykol, the *B. mori* pheromone bearing the same hydroxyl headgroup but differing in double-bond positions ((10*E*,12*Z*) versus (6*E*,11*Z*)), showed a slightly higher affinity (−5.3 kcal/mol) than the metabolite (Table S1). These results indicate that while headgroup polarity dominates affinity, the position of double bonds also modulates ligand conformation and hydrophobic contacts with residues such as leucine, valine, and isoleucine.

Taken together with the docking data, these observations illustrate that ApolPBP1 binding is governed by a delicate interplay of steric complementarity, hydrophobic packing, and polar interactions. Even subtle modifications in chain length, headgroup chemistry, or unsaturation pattern can significantly alter the binding affinity and ligand specificity (Table S1).

Despite these computational predictions of comparable binding energies for the pheromone and the analog, experimental data revealed a 4-fold lower affinity for the analog compared to the natural pheromone. This discrepancy likely reflects the inherent limitations of docking approaches, including inadequate solvation modeling, restricted protein flexibility, and limited conformational sampling. These findings underscore the importance of integrating computational predictions with experimental validation, particularly for dynamic systems, such as PBPs.

## Conclusion

Our findings underscore the importance of structural precision in the design of pheromone analogs. The reduced affinity of the elongated hydrophobic analog, despite increased hydrophobicity, illustrates the delicate balance between enhancing hydrophobic contacts and maintaining critical molecular interactions within the binding pocket. Future analog design should retain key features of the natural pheromone while optimizing hydrophobic and steric complementarity.

## Supplementary Material



## Notes

Experimental details for the synthesis of the pheromone analog, (6*E*,11*Z*)-heptadeca-6,11-dien-1-yl acetate, and characterization by HRMS and NMR; fluorescence data of pheromone binding assay; docking and LigPlot of related compounds; and binding interactions (PDF)
