# Supplementary material for: Disrupting Pheromone Signaling in Insects: Design, Synthesis, and Evaluation of an Inhibitor
Source: ACS Omega. 2026 Jan 16;11(8):13052–61. doi: 10.1021/acsomega.5c06376 (PMC12961486; doi:10.1021/acsomega.5c06376)
Supplement: Supplementary file 1 [file ao5c06376_si_001.pdf]

Supporting Information

Disrupting Pheromone Signaling in Insects: Design, Synthesis and Evaluation of an Inhibitor

Pratikshya Paudel[1], Ishani Ray[1], Omar Al Danoon[1], James R. Howard[2], Josef M. Maier[2], Sarah R. Moor[2], Anna M. Lidskog[2], Eric V. Anslyn[2], Smita Mohanty[1*]

[1]Department of Chemistry, Oklahoma State University, Stillwater, Oklahoma 74078, United States.

[2]Department of Chemistry, University of Texas at Austin, Austin, Texas 78 712, United States.

*Corresponding author: Smita Mohanty, Department of Chemistry, Oklahoma State University, Stillwater, Oklahoma 74078, United States; Email: smita.mohanty@okstate.edu

Table of Content

Synthesis and Characterization of Pheromone Analog (Figure S1-S15)……………………..S1-S16

Competitive fluorescence binding assay (Figure S16)………………..……..……………..……….S17

Ligplot diagram for ApolPBP1-ligand complex (Figure S17)……….....…………………….…..S18

Docking predicted binding interactions and energies (Table S1)……..………...……………..S19

Experimental Details

Synthesis and Characterization of Pheromone Analog

All reactions were performed under a nitrogen atmosphere with anhydrous solvent unless otherwise stated. All reagents were purchased from commercial suppliers and used without further purification. ^{1}H and ^{13}C NMR spectra were obtained on a 500 MHz Bruker spectrometer equipped with a 5mm high sensitivity nitrogen two channel Prodigy cryo-probe or a 400 MHz Agilent MR400 spectrometer. All NMR spectra were acquired in deuterated solvents purchased from Cambridge Isotope Laboratories, Inc. Chemical shifts are reported in ppm and referenced to the solvent peak. High-resolution mass spectrometry (HRMS) data were collected using an Agilent 6530 Accurate-Mass Q-TOF LC/MS or Agilent 6546 Q-TOF LC/MS (UT Austin Mass Spectrometry Facility).

2-(Pent-4-yn-1-yloxy) tetrahydro-2*H*-pyran (2)

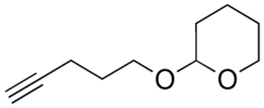

To a 50 mL round bottom flask was added 4-pentyne-1-ol (10 mL, 112.2 mmol, 1 eq) 3,4-dihydro-2H-pyran (9.43 g, 112.17 mmol, 1 eq), and dichloromethane (30 mL). The reaction mixture was cooled to 0 ºC with an ice bath. Camphor sulfonic acid (47 mg, 0.202 mmol) was added. The ice bath was removed, and the reaction was allowed to stir overnight at room temperature. The reaction was quenched with saturated $NaHCO_3$ (30 mL) and extracted with DCM (3 x 10 mL). The organic layers were combined and dried with $MgSO_4$, then concentrated under reduced pressure to yield **2** as a pale-yellow oil (16.5 g, 88% yield). ^{1}H NMR (400 MHz, $CDCl_3$) δ 4.62–4.51 (m, 1H), 3.90–3.74 (m, 2H), 3.54–3.41 (m, 2H), 2.29 (tdd, J = 6.9, 2.7, 0.9 Hz, 2H), 1.92 (t, J = 2.7 Hz, 1H), 1.85–1.75 (m, 3H), 1.73–1.65 (m, 1H), 1.59–1.48 (m, 4H).

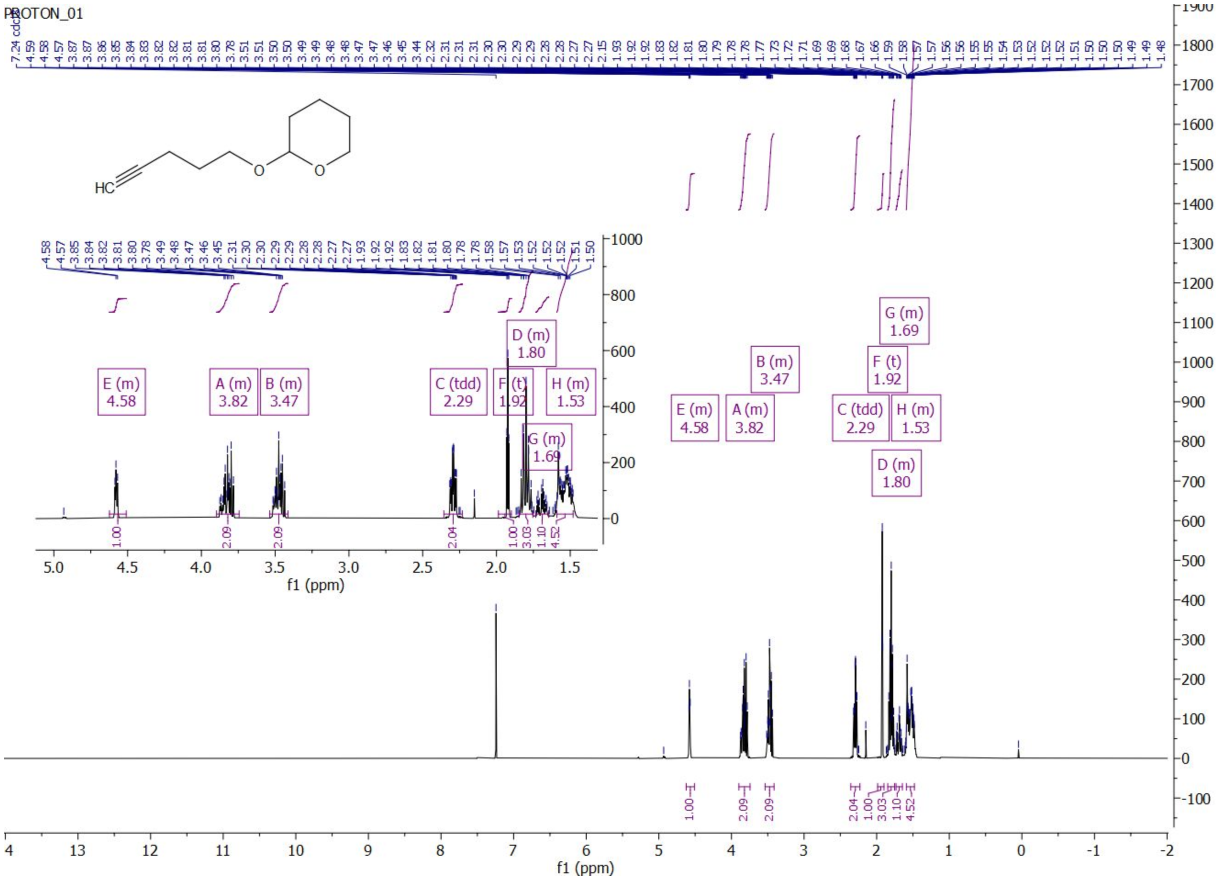

Figure S1. ^{1}H NMR spectrum (400 MHz, $CDCl_3$) of **2**.

2-(Dec-4-yn-1-yloxy)tetrahydro-2*H*-pyran (3)

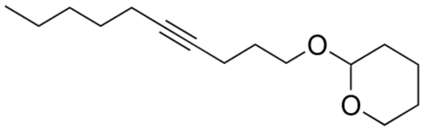

To a 100 mL Schlenk flask was added **2** (3.28 g, 25.0 mmol, 1 eq) and anhydrous THF (30 mL). The solution was cooled to -78 ºC in an acetone-dry ice bath. *n*-BuLi (excess) was added slowly over five minutes. 1-Iodopentane (3.8 mL, 29.3 mmol, 1.2 eq) was added and the reaction mixture was allowed to warm to room temperature. The reaction was refluxed for 16 h, after which it was allowed to cool to room temperature. The reaction mixture was diluted with 1M NH_4Cl (30 mL) and extracted with hexanes (3 x 10 mL). The combined organic layers were dried over $NaSO_4$ and concentrated under reduced pressure, giving **3** as a clear oil (4.23 g, 71% yield). ^{1}H NMR (400 MHz, $CDCl_3$) δ 4.61–4.55 (m, 1H), 3.90–3.75 (m, 2H), 3.53–3.40 (m, 2H), 2.25 (tq, *J* = 7.0, 2.0 Hz, 2H), 2.11 (tt, *J* = 7.1, 2.4 Hz, 2H), 1.86–1.63 (m, 4H), 1.62–1.22 (m, 10H), 0.92–0.83 (m, 3H).

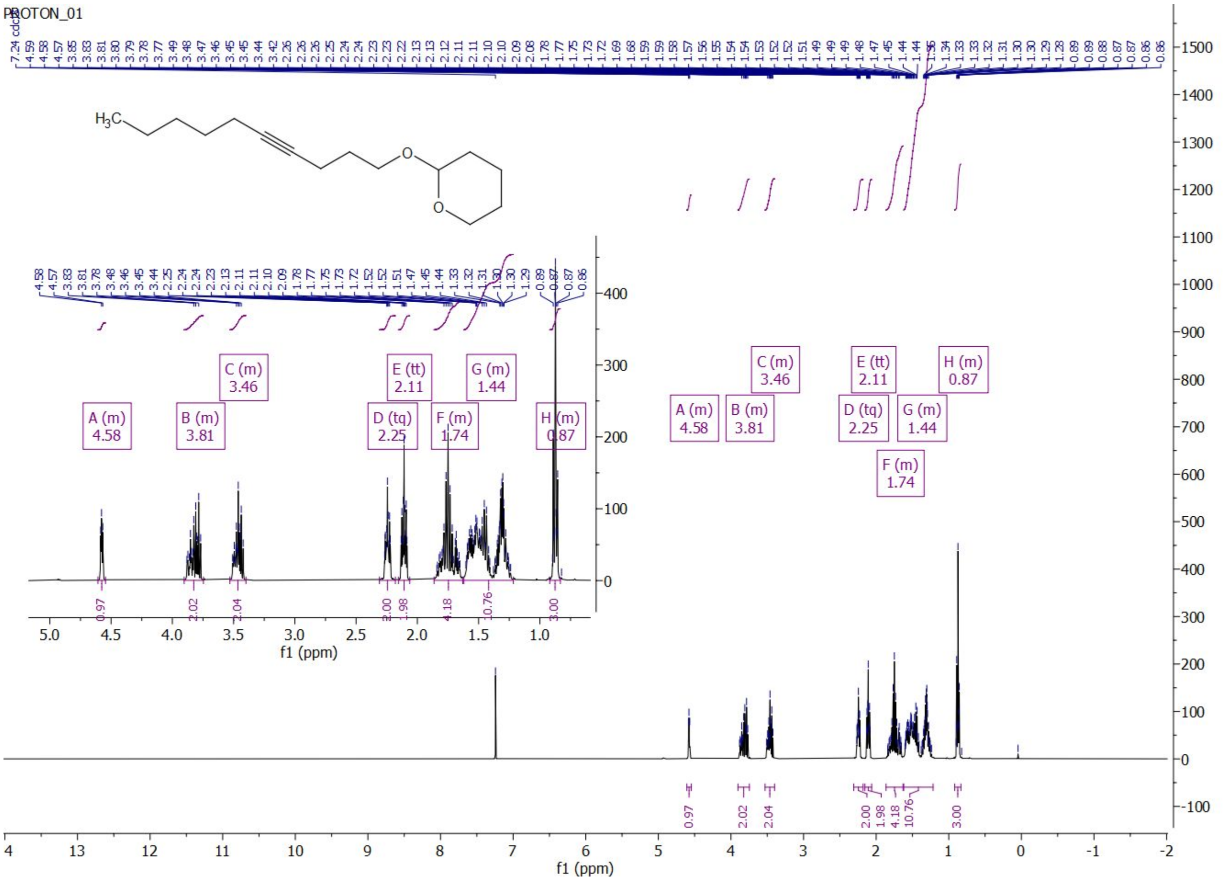

Figure S2. ^{1}H NMR spectrum (400 MHz, $CDCl_3$) of **3**.

Dec-4-yn-1-ol (4)

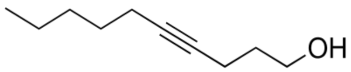

To a 100 mL round bottom flask was added **3** (7.52 g, 31.6 mmol, 1 eq), *p*-toluenesulfonic acid (0.335 g, 1.95 mmol) and methanol (30 mL). The reaction was stirred overnight at room temperature, then poured into 0.5 M NaOH (50 mL). The mixture was extracted with hexanes (3 x 30 mL) and concentrated under reduced pressure. The product was purified by flash column chromatography (hexanes) to yield **4** as a clear oil, (3.77 g 84% yield). ^{1}H NMR (400 MHz, $CDCl_3$) δ 3.74 (t, *J* = 6.1 Hz, 2H), 2.26 (tt, *J* = 6.8, 2.4 Hz, 2H), 2.12 (tt, *J* = 7.1, 2.4 Hz, 2H), 1.72 (p, *J* = 6.5 Hz, 2H), 1.45 (h, *J* = 6.5 Hz, 2H), 1.32 (ddt, *J* = 11.7, 8.7, 4.4 Hz, 4H), 0.88 (t, *J* = 7.1 Hz, 3H).

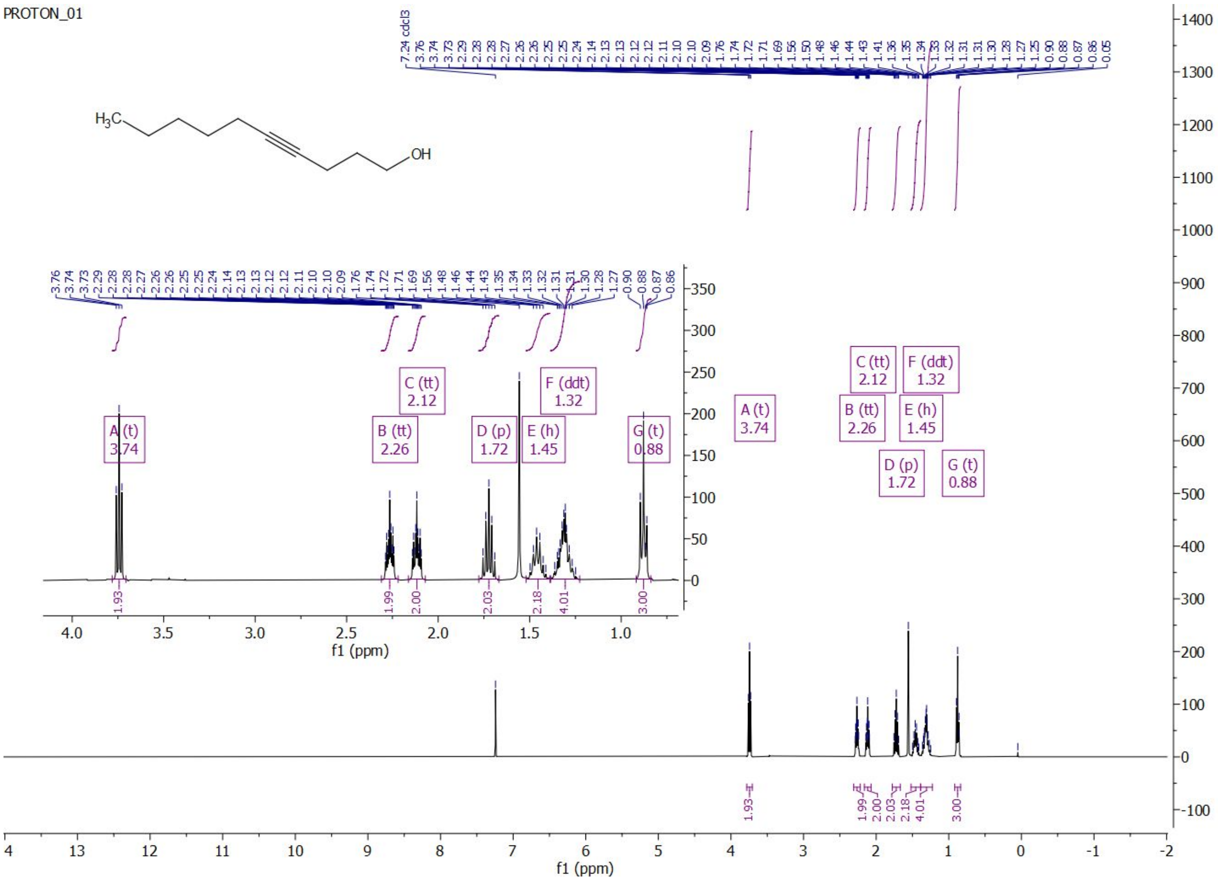

Figure S3. ^1H NMR spectrum (400 MHz, $CDCl_3$) of **4**.

(4*Z*)-Dec-4-en-1-ol (5)

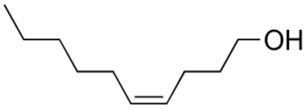

To a 250 mL round bottom flask was added a solution of $Ni(OAc)_2·4H_2O$ in EtOH (100 mL, 80.4 mM, 8.04 mmol, 0.34 eq). The flask was sealed and purged of air. A balloon of H_2(g) was attached. In a separate flask, $NaBH_4$ (800 mg, 21.1 mmol) and NaOH (aq., 2M, 1 mL) were dissolved in EtOH (19 mL). Under vigorous stirring, $NaBH_4$ solution (2 mL, 2.1 mmol) was added dropwise to the $Ni(OAc)_2·4H_2O$ solution to produce a black suspension. The reaction was stirred for 10 minutes, after which ethylene diamine (1.6 mL, 24 mmol, 1 eq) was added and stirred for an additional 10 minutes. **4** (3.7 g, 24.0 mmol, 1 eq) was added and the reaction was stirred at room temperature overnight. The residual hydrogen was flushed with N_2(g) and the reaction mixture was filtered through a thin pad of celite and washed with EtOH. The reaction was concentrated under reduced pressure to approximately 20 mL total volume and poured into 1M HCl (200 mL). The aqueous mixture was extracted with hexanes (3 x 20 mL) and the organic fractions were combined and dried over Na_2SO_4. The organic fractions

were concentrated to yield **5** as a pale-yellow oil (2.7 g, 72% yield). ^{1}H NMR (400 MHz, $CDCl_3$) δ 5.38 (d, *J* = 6.3 Hz, 2H), 3.65 (s, 2H), 2.16–1.99 (m, 4H), 1.63 (s, 2H), 1.29 (s, 6H), 0.88 (s, 3H).

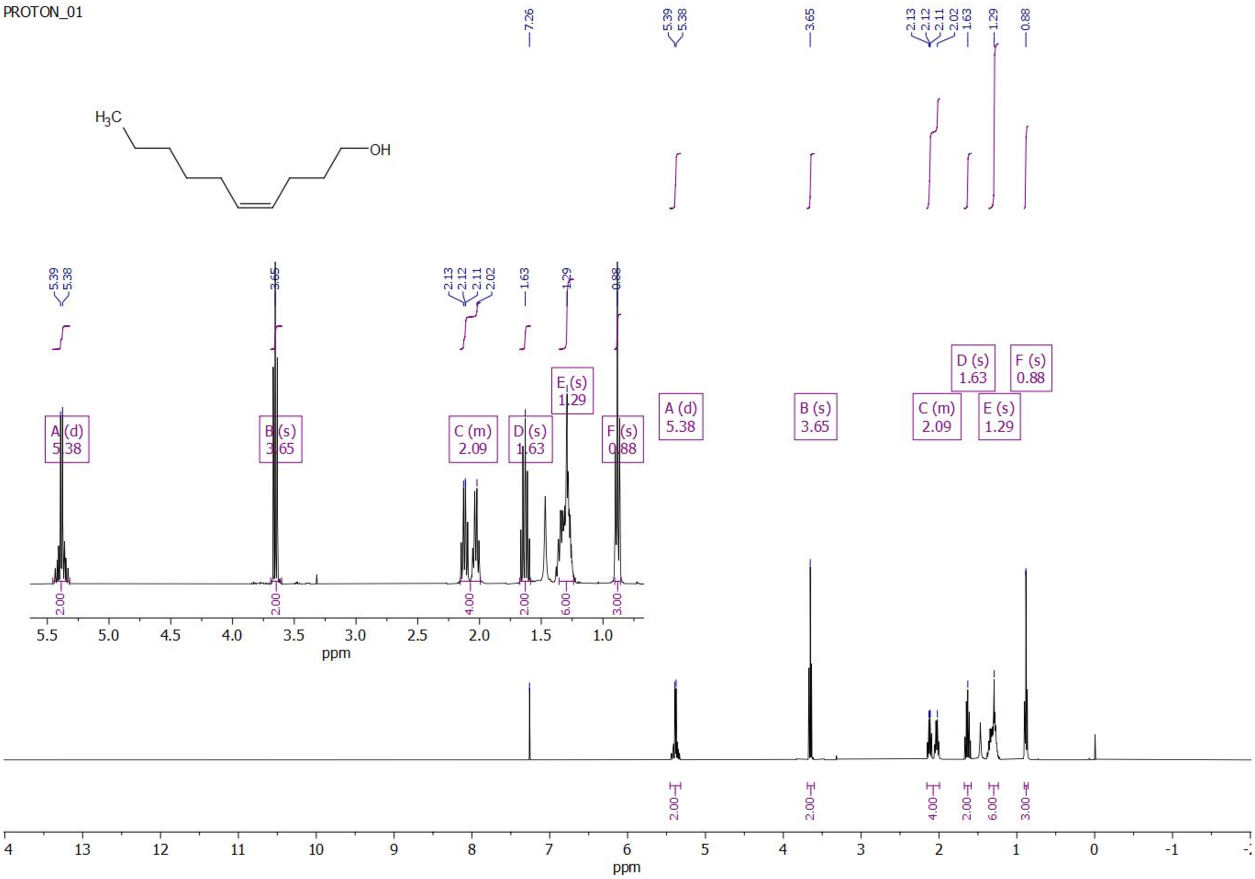

Figure S4. ^{1}H NMR spectrum (400 MHz, $CDCl_3$) of **5**.

(4*Z*)-1-Bromodec-4-ene (6)

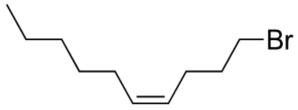

5 (3.0 g, 19.1 mmol, 1 eq) and Et_3N (2.5 g, 24.83 mmol, 1.3 eq) were dissolved in DCM. The vial was cooled in an ice bath to 0 ºC. Methanesulphonyl chloride (2.4 g, 21.01 mmol, 1.1 eq) was added dropwise. The resulting slurry was stirred at 0 ºC for 2.5 h, then poured into ice-water (50 mL). The organic layer was separated. The aqueous layer was extracted with DCM (5 mL). The organic layers were combined and washed with 1 M HCl (10 mL), saturated $NaHCO_3$ (10 mL), and brine (10 mL). The organic layer was then dried with Na_2SO_4 and concentrated under reduced pressure. The residue was dissolved in acetone (7 mL) and placed into a 6-dram vial. LiBr (5 g, 57.5 mmol, 3 eq) was added, and the mixture was sealed with a cap and stirred at 50 °C overnight, by which time all the mesylate had been consumed. The slurry was cooled and poured into deionized water (50 mL), then extracted

with hexanes. The hexane layer was washed with brine, dried with Na_2SO_4, and concentrated under reduced pressure to yield **6** as a pale yellow oil (1.84 g, 44% yield). ^{1}H NMR (500 MHz, $CDCl_3$) δ 5.45 (ddt, *J* = 10.8, 8.7, 4.3 Hz, 1H), 5.35–5.26 (m, 1H), 3.41 (t, *J* = 6.7 Hz, 2H), 2.20 (q, *J* = 7.2 Hz, 2H), 2.05 (q, *J* = 7.0 Hz, 2H), 1.91 (p, *J* = 6.8 Hz, 2H), 1.31 (tdd, *J* = 15.1, 12.9, 5.6 Hz, 6H), 0.89 (t, *J* = 6.9 Hz, 3H).

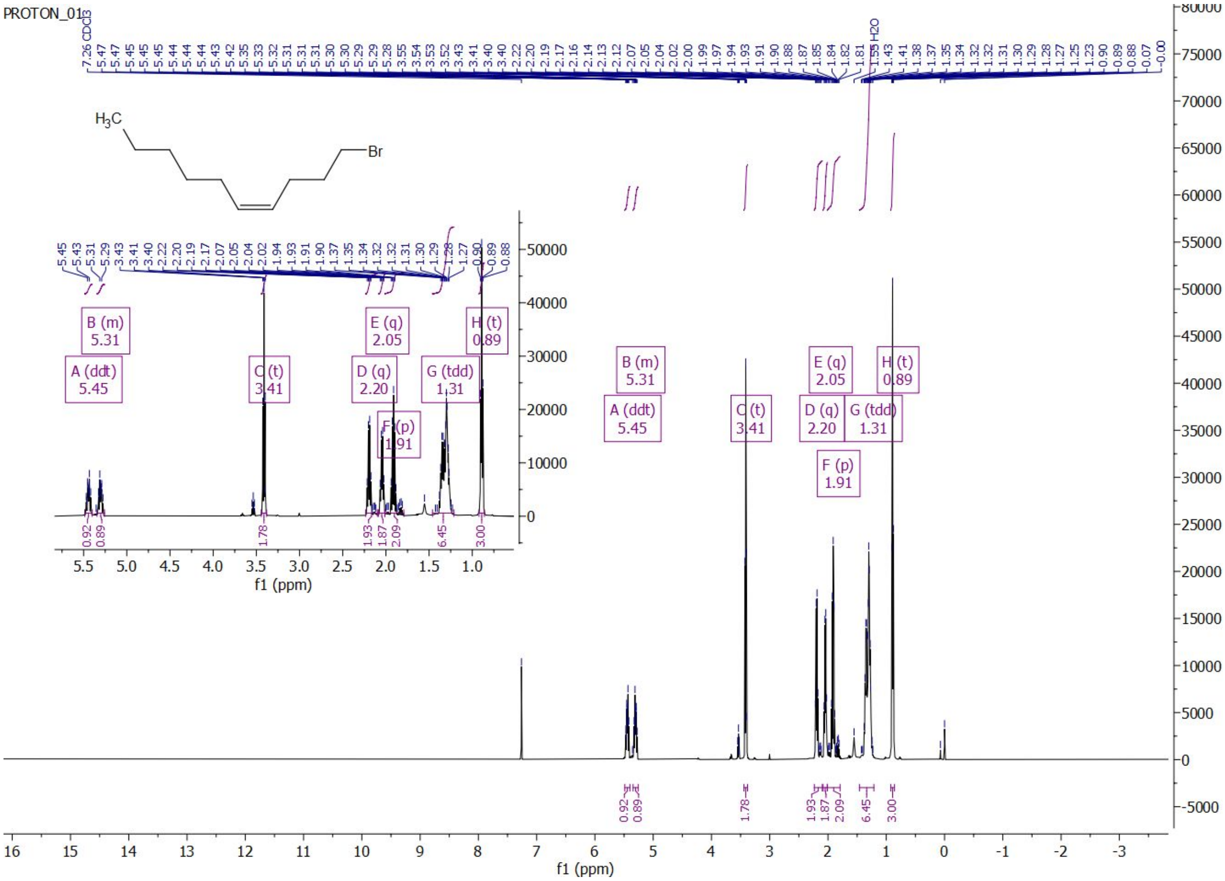

Figure S5. ^{1}H NMR spectrum (500 MHz, $CDCl_3$) of **6**.

2-(Hept-6-yn-1-yloxy)tetrahydro-2*H*-pyran (7)

To a 50 mL round bottom flask was added hept-6-yn-1-ol (5.00 g, 44.6 mmol, 1 eq), 3,4-dihydro-2H-pyran, (4.13 g, 49.1 mmol, 1.1 eq) and DCM (30 mL). The reaction mixture was cooled to 0 ºC with an ice bath. Camphor sulfonic acid (47 mg, 0.202 mmol) was added. The ice bath was removed, and the reaction was allowed to stir overnight at room temperature. The reaction was quenched with saturated $NaHCO_3$ (30 mL) and extracted with DCM (3 x 10 mL). The organic layers were combined and dried with $MgSO_4$, then concentrated under reduced pressure to yield **7** as a pale-yellow oil (7.69 g, 88% yield). ^{1}H NMR (500 MHz, $CDCl_3$) δ 4.53–4.48 (m, 1H), 3.80

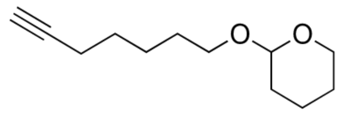

(ddd, J = 11.0, 7.8, 3.0 Hz, 1H), 3.68 (dt, J = 9.6, 6.8 Hz, 1H), 3.47–3.39 (m, 1H), 3.32 (dt, J = 9.6, 6.6 Hz, 1H), 2.13 (td, J = 7.0, 2.6 Hz, 2H), 1.87 (t, J = 2.6 Hz, 1H), 1.68–1.34 (m, 12H).

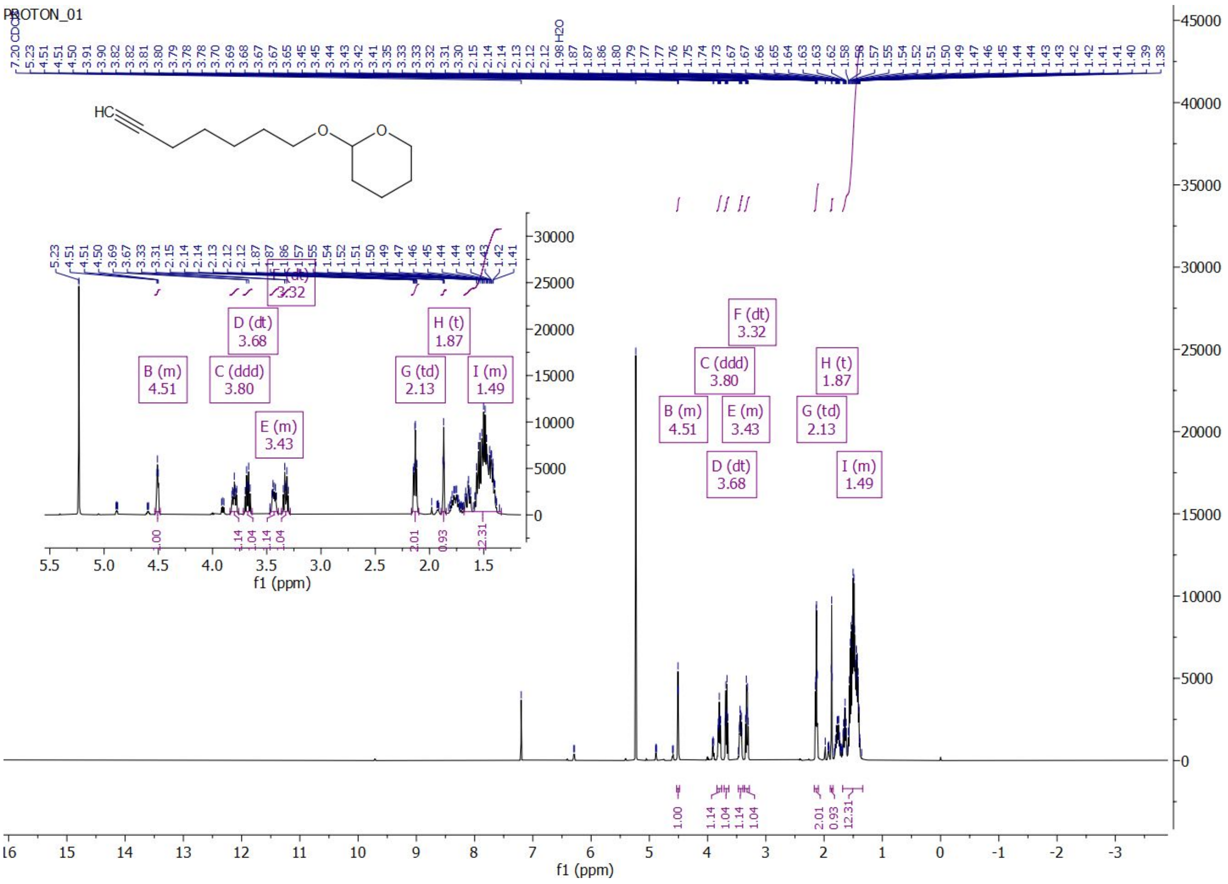

Figure S6. ^{1}H NMR spectrum (500 MHz, $CDCl_3$) of **7**.

(11*Z*)-2-(Heptadec-11-en-6-yn-1-yloxy)tetrahydro-2*H*-pyran (9)

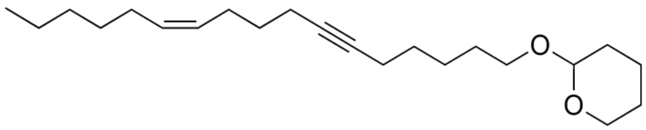

A flame dried vial equipped with a stir bar was sealed with rubber septum and secured with copper wire. The vial was purged of air and equipped with an argon balloon. A solution of **8** (729 mg, 3.71 mmol, 1 eq) in hexane (0.5 mL) was added. The vial was cooled to 0 °C in an ice bath. Under vigorous stirring, *n*-BuLi (2 M in cyclohexane, 1.98 mL, 3.96 mmol, 1.1 eq) was added dropwise over 5 minutes. The solution was stirred at 0 °C for an additional 10 minutes and then allowed to warm to room temperature. A solution of **6** (835 mg, 3.81 mmol, 1 eq) in anhydrous HMPA (1 mL) was added dropwise while maintaining a temperature slightly below room temperature using an ice bath intermittently. The

reaction vessel was removed from the ice bath and stirred vigorously at room temperature for 20 hours, after which the solution was poured into ice water (50 mL). The mixture was extracted with Et_2O (5 x 5mL). The ether extracts were combined and washed with brine (10 mL), then concentrated under reduced pressure. The final product was purified with flash column chromatography (hexanes) to afford **9** as a clear oil. ^{1}H NMR (500 MHz, $CDCl_3$) δ 5.45–5.23 (m, 2H), 4.57–4.52 (m, 1H), 3.83 (ddd, J = 11.0, 7.8, 3.1 Hz, 1H), 3.71 (dt, J = 9.6, 6.7 Hz, 1H), 3.53–3.43 (m, 1H), 3.41–3.32 (m, 2H), 2.24–1.95 (m, 6H), 1.92–1.67 (m, 4H), 1.60–1.43 (m, 10H), 1.27 (ddd, J = 15.2, 10.1, 5.7 Hz, 7H), 0.86 (t, J = 6.7 Hz, 3H).

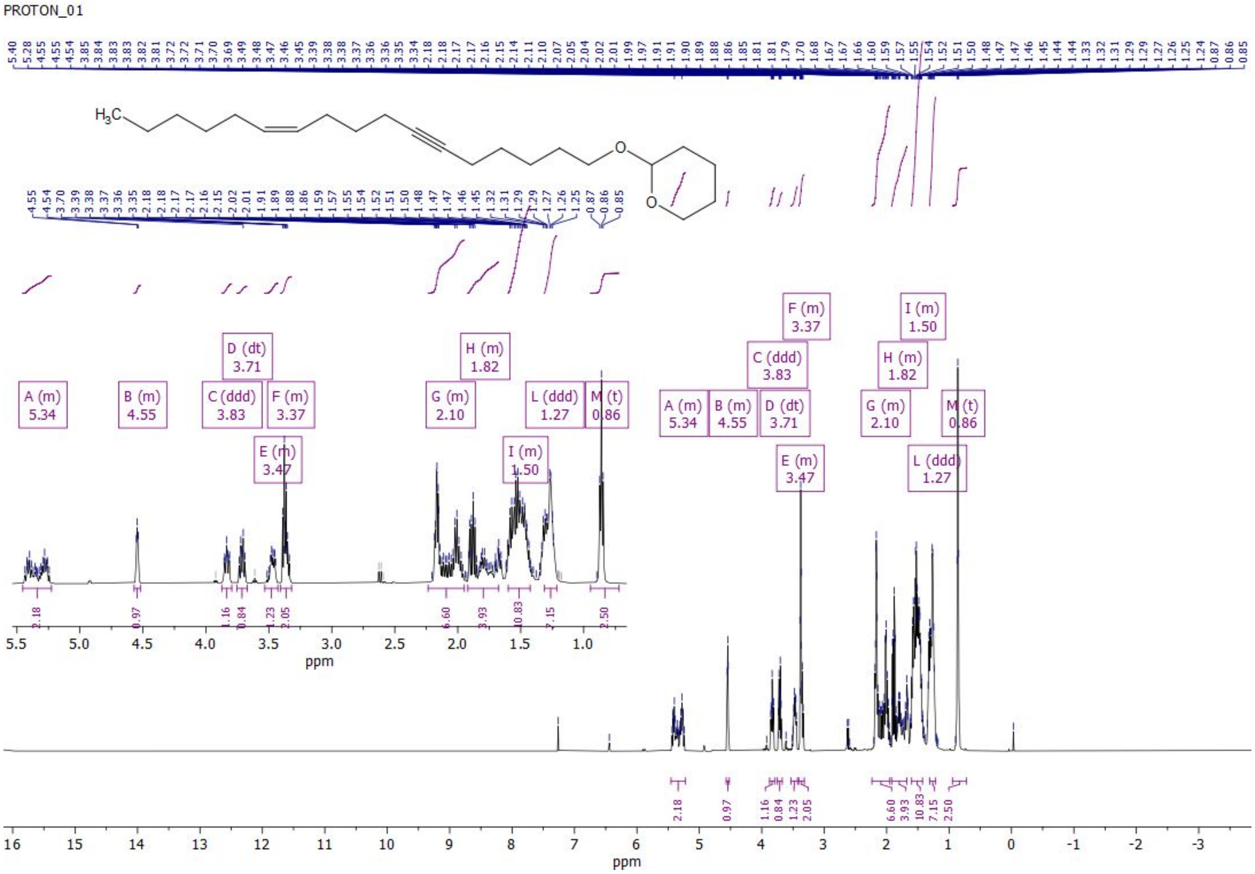

Figure S7. ^{1}H NMR spectrum (500 MHz, $CDCl_3$) of **9**.

(11*Z*)-Heptadec-11-en-6-yn-1-ol (10)

A flame dried 6-dram vial was charged with **9** (292 mg, 0.87 mmol, 1 eq) and equipped with a stir bar. The flask was sealed with septum, purged of air, and backfilled with N_2 three times. A solution of *p*-toluenesulfonic acid (10 mg) in anhydrous methanol (5 mL) was added to the reaction flask at room temperature under vigorous stirring. The reaction was stirred at room temperature for 3

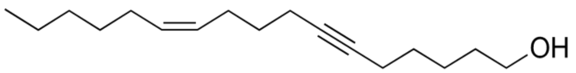

hours, after which it was quenched with saturated aqueous $NaHCO_3$ (15 mL). The residue was extracted with Et_2O (3 x 20 mL), and the organic layers were combined and washed with brine (15 mL). The organic layer was dried over Na_2SO_4 and concentrated under reduced pressure. The resulting residue was used in the next step without purification (184 mg, 84% yield).

(6*E*,11*Z*)-Heptadeca-6,11-dien-1-ol (11)

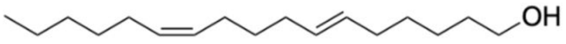

Sodium metal (100 mg, 4.34 mmol) was washed with hexane and transferred into a 50 mL flame dried schlenk flask equipped with a magnetic bar and a septum. The reaction flask was cooled to –78 °C using a dry ice/acetone bath and NH_3 gas was condensed into the reaction flask until a dark blue solution (around 30 mL) was formed. While stirring, a solution of **10** (184 mg, 0.73 mmol) in THF (1 mL) was added dropwise into the dark blue solution at –78 °C. Stirring was continued at –78 °C for 3 h, after which an additional small portion of sodium was added and stirred for another 1 h. *t*-Butanol was added slowly, causing gas evolution. The reaction flask was opened to air and brought to room temperature. The reaction was stirred at room temperature for an additional 30 minutes, after which sufficient *tert*-butanol was added to quench any remaining metal. After NH_3 was removed under reduced pressure, DCM (5 mL) was added and the mixture was stirred for 30 min. The reaction mixture was extracted with DCM and hexanes. The solvent was removed under reduced pressure and the obtained crude **11** was used in the next step without further purification. ^{1}H NMR (500 MHz, $CDCl_3$) δ 5.36 (ddt, *J* = 26.7, 11.0, 4.6 Hz, 3H), 3.64 (q, *J* = 6.6 Hz, 2H), 2.20–2.08 (m, 2H), 2.01 (dq, *J* = 15.2, 8.5 Hz, 5H), 1.62–1.20 (m, 16H), 0.89 (t, *J* = 6.9 Hz, 3H).

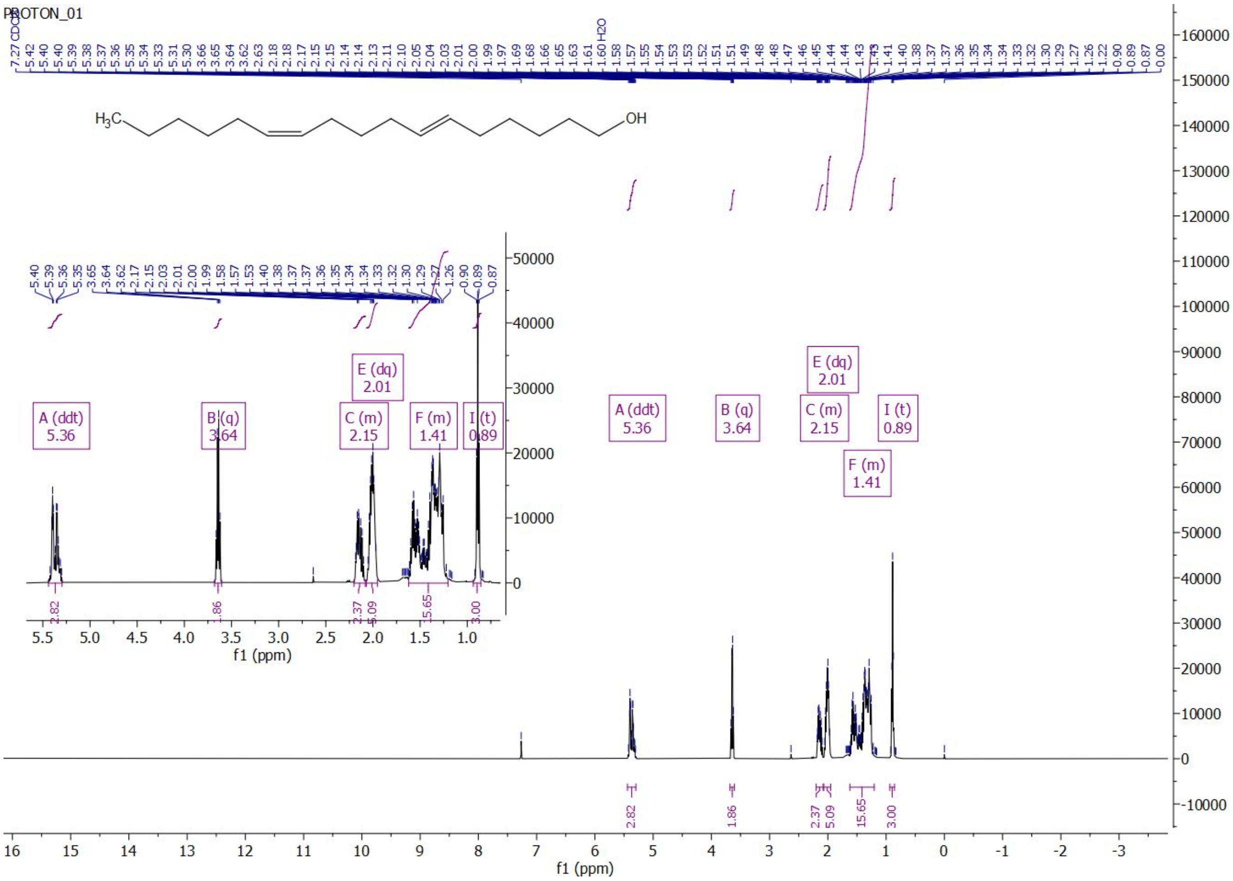

Figure S8. ^{1}H NMR spectrum (500 MHz, $CDCl_3$) of **11**.

(6*E*,11*Z*)-heptadeca-6,11-dien-1-yl acetate (12)

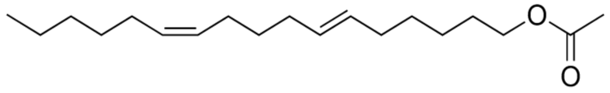

To a flame dried vial was added **11** (102 mg, 0.40 mmol, 1 eq) and anhydrous THF (2.5 mL). A solution of DMAP (catalytic amount) in anhydrous THF (0.5 mL) was added. Et_3N (52.5 µL, 0.38 mmol, 1 eq) and Ac_2O (35 µL, 0.38 mmol, 1 eq) were added and the mixture was stirred at room temperature for 4 h. The reaction was cooled to 0 °C and deionized H_2O (5 mL) was added. The mixture was extracted with hexane (3 × 10 mL) and the combined organic layers were washed with brine (15 mL), dried over Na_2SO_4, and concentrated under reduced pressure. The residue was purified by flash chromatography on silica gel (hexane) to afford stereoisomerically pure acetate **12** as a colorless oil (112 mg, 95%). ^{1}H NMR (500 MHz, $CDCl_3$) δ 5.41–5.37 (m, 2H), 5.37–5.30 (m, 2H), 4.05 (t, *J* = 6.7 Hz, 2H), 2.04 (s, 3H), 2.04–1.96 (m, 8H), 1.62 (p, *J* = 6.8 Hz, 2H), 1.41–1.25 (m, 12H), 0.88 (t, *J* = 6.9 Hz, 3H). ^{13}C NMR (126 MHz, $CDCl_3$) δ 171.3, 130.6, 130.3, 129.6, 64.7, 32.5, 32.3, 31.7, 29.8, 29.6, 29.3, 28.6, 27.3, 26.8, 25.5 22.7, 21.1, 14.2.

HRMS *m/z* **calcd** for $C_{19}H_{34}O_2$ $[M+Na]^+$: 317.2451, found 317.2453

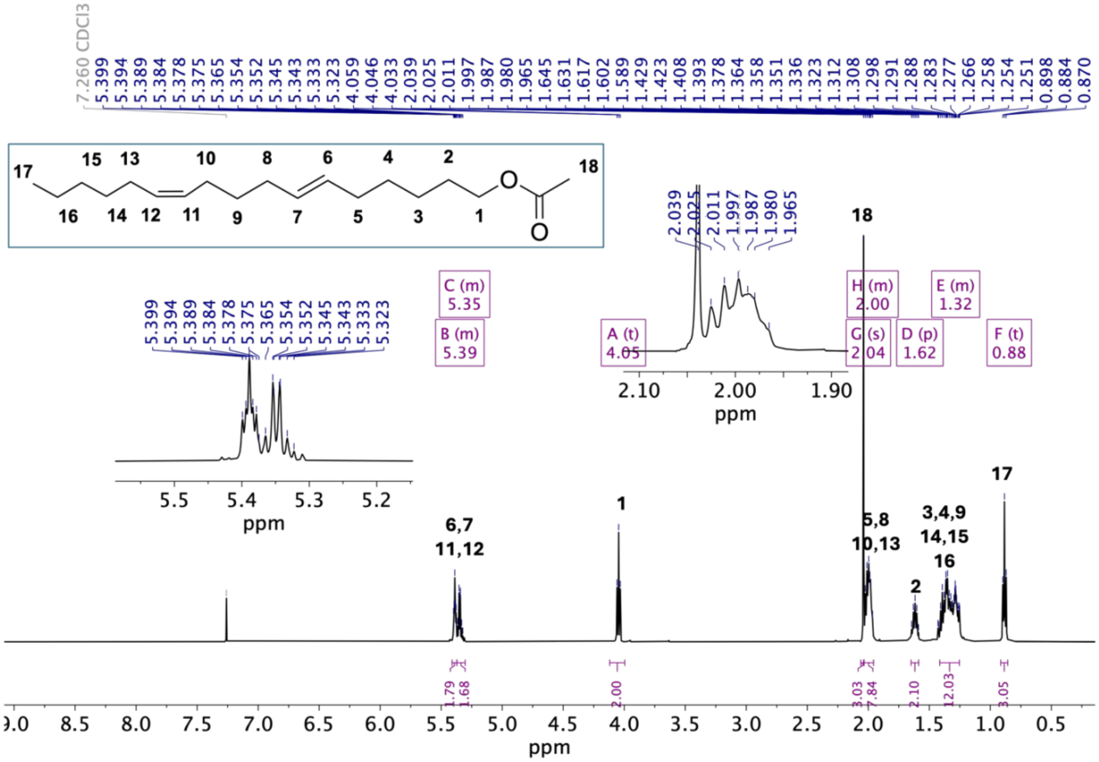

Figure S9. ^{1}H NMR spectrum (500 MHz, $CDCl_3$) of **12**. Assignment based on ^{1}H NMR and COSY.

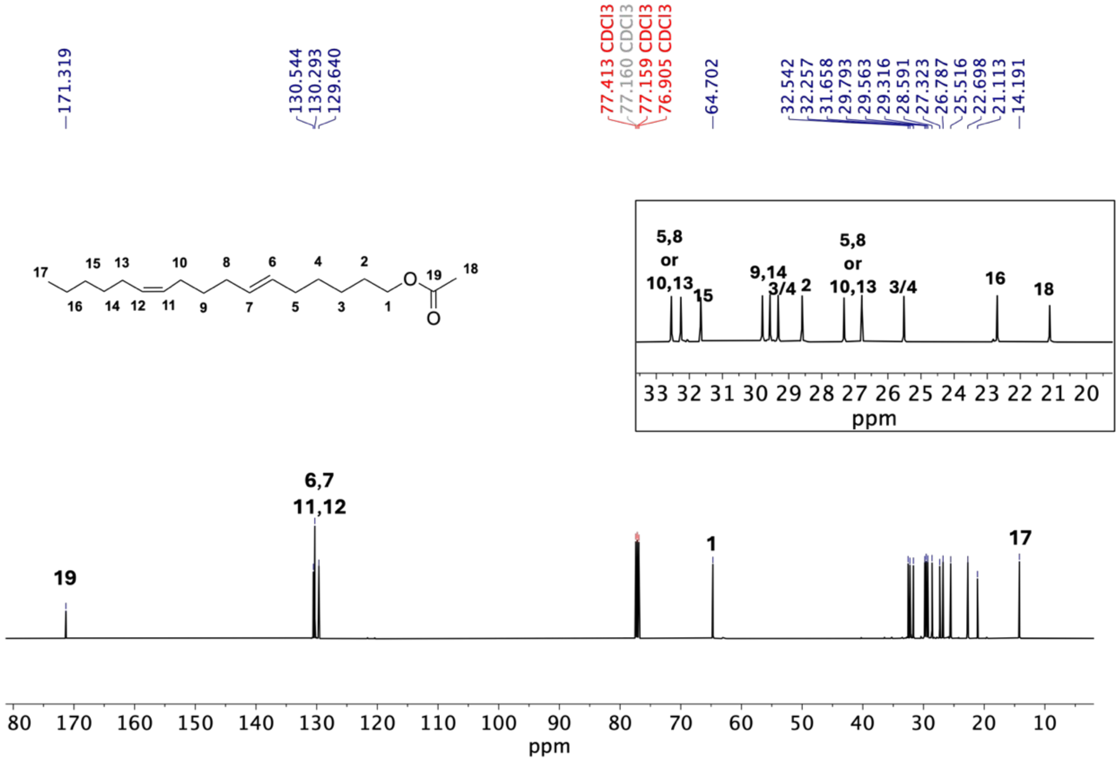

Figure S10. ^{13}C NMR spectrum (126 MHz, $CDCl_3$) of **12**. Assignment based on ^{1}H, ^{13}C, COSY, HSQC and HMBC.

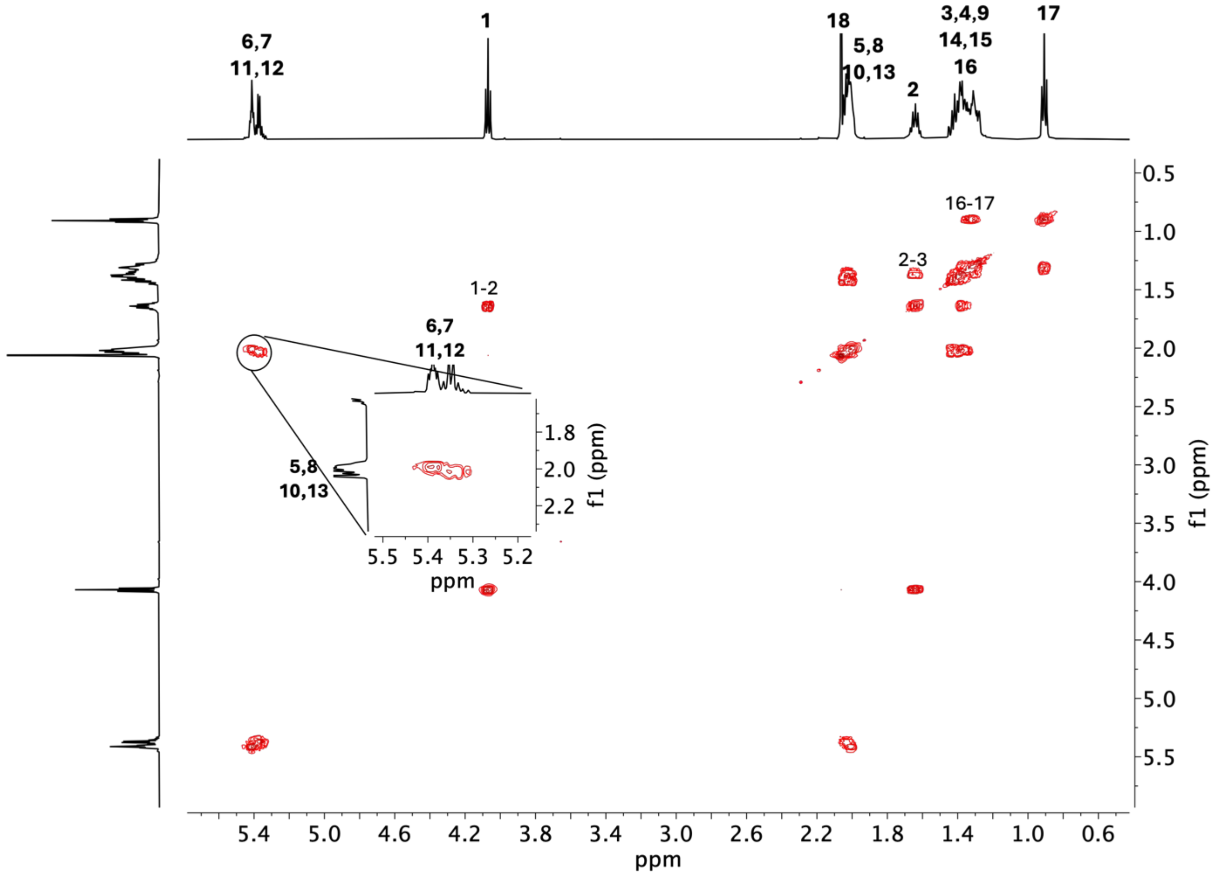

Figure S11. COSY of **12**. Cross-peaks between H1-H2, H2-3, and H16-H17 are marked. The inset shows the cross-peaks between the alkene protons (H6, H7, H11, H12) and their respective neighboring methylene protons (H5, H8, H10, H13).

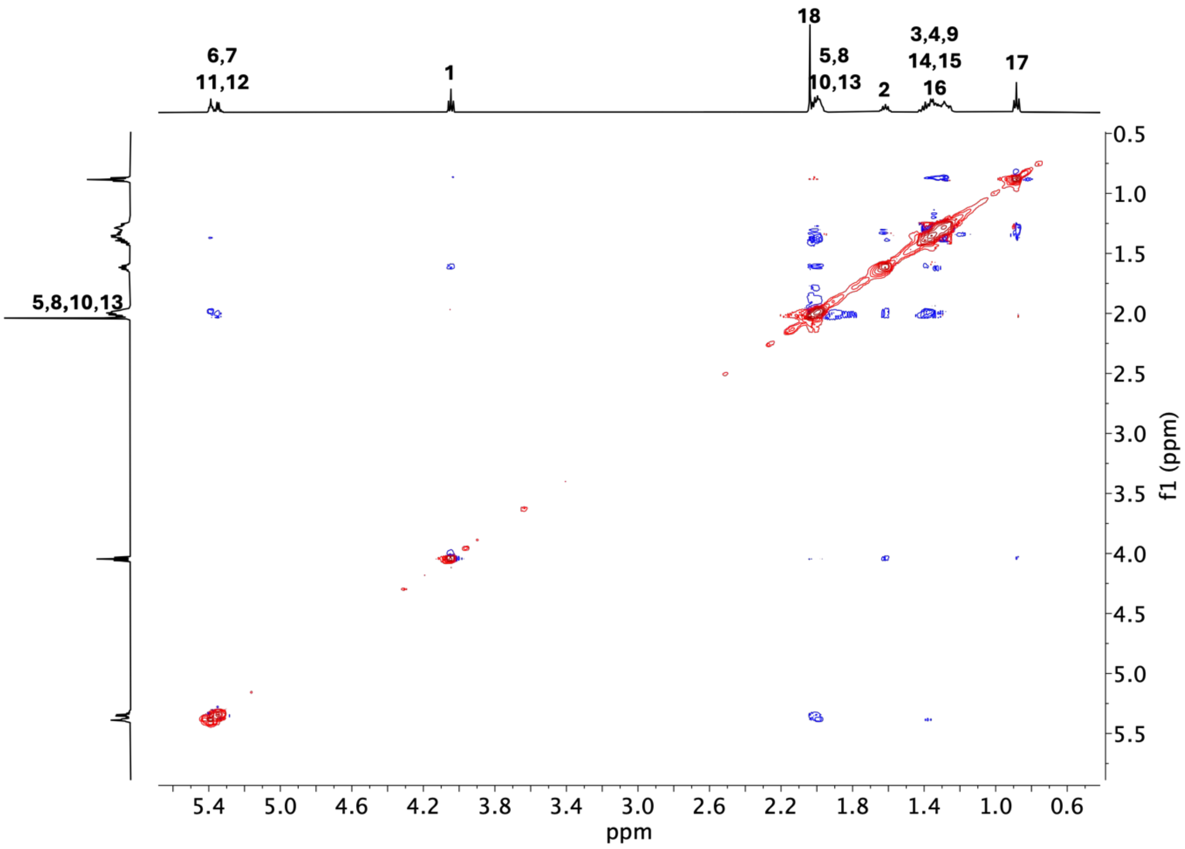

Figure S12. NOESY of **12** in $CDCl_3$ (800 ms mixing time). NOESY recorded with both 300 and 800 ms mixing time showed very weak/no NOEs. This is most likely due to the high flexibility of **12**, where most parts of the molecule are in close proximity to each other at some point, leading to the interactions averaging out on the NMR time scale. In theory, the methylene protons on each side of a *cis* alkene are expected to have an NOE, while the methylene protons on each side of a *trans* alkene are too far apart and therefor will not show any NOE (Figure S13). However, due to the overlap of the chemical shifts and the high flexibility of the molecule, the presence/absence of such NOEs could not be observed for **12**.

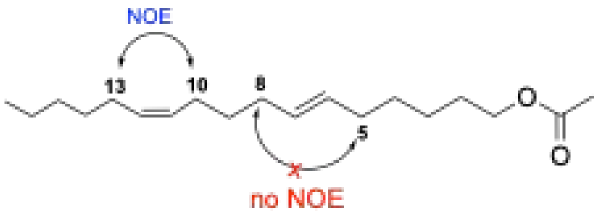

Figure S13. Expected NOEs in **11**.

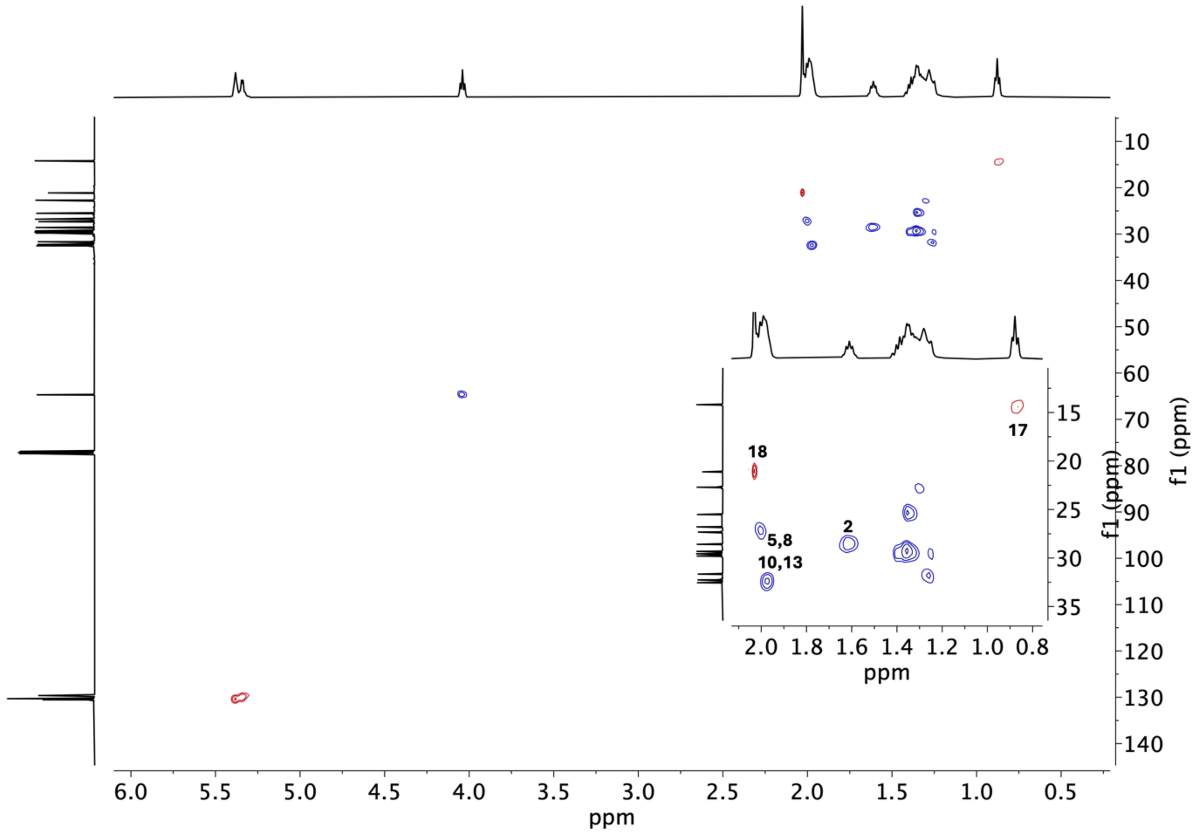

Figure S14. HSQC of **12** in $CDCl_3$.

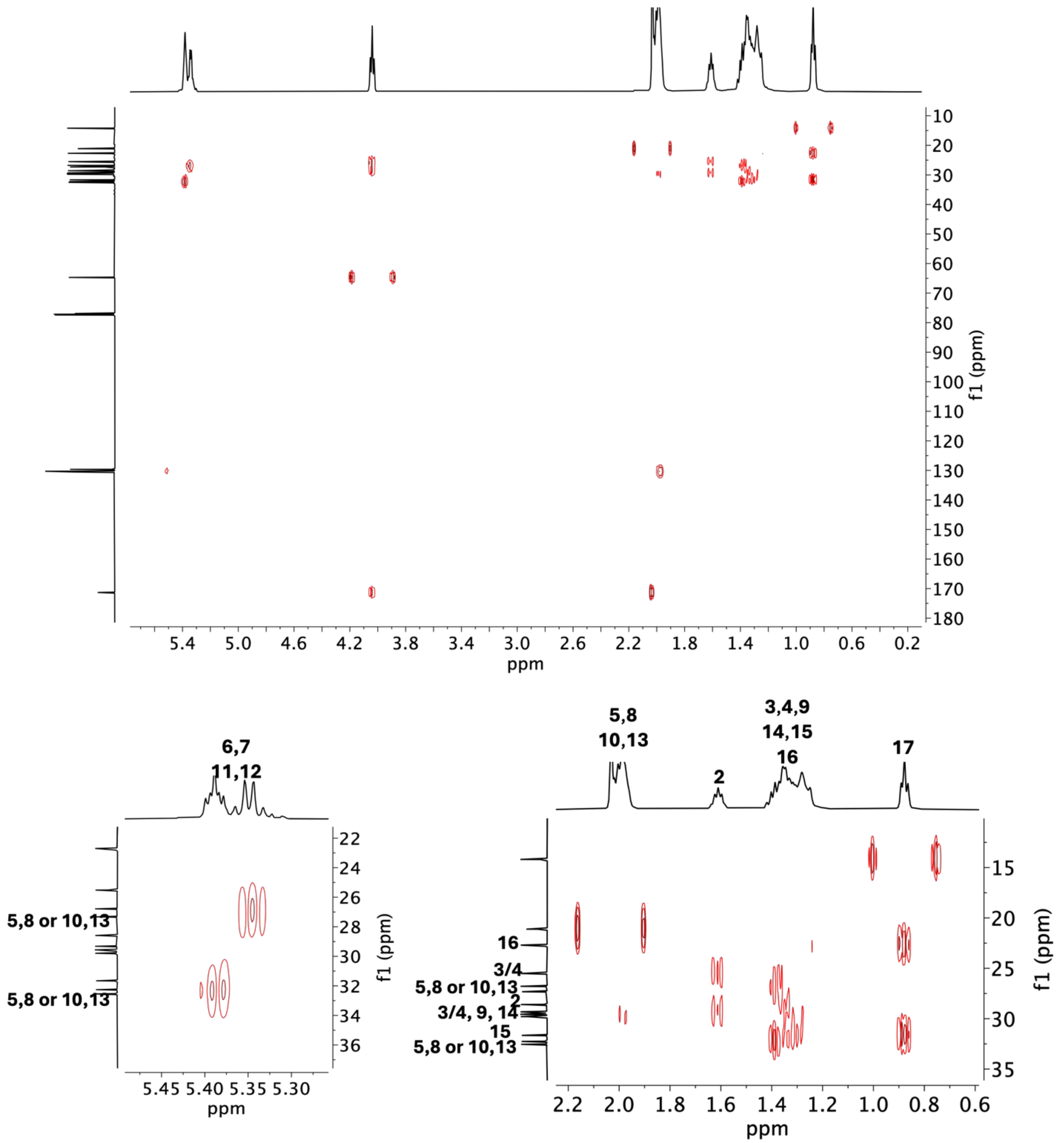

Figure S15. HMBC of **12** in $CDCl_3$. Full spectrum (top) and zoomed in sections (bottom).

Competitive fluorescence binding assay

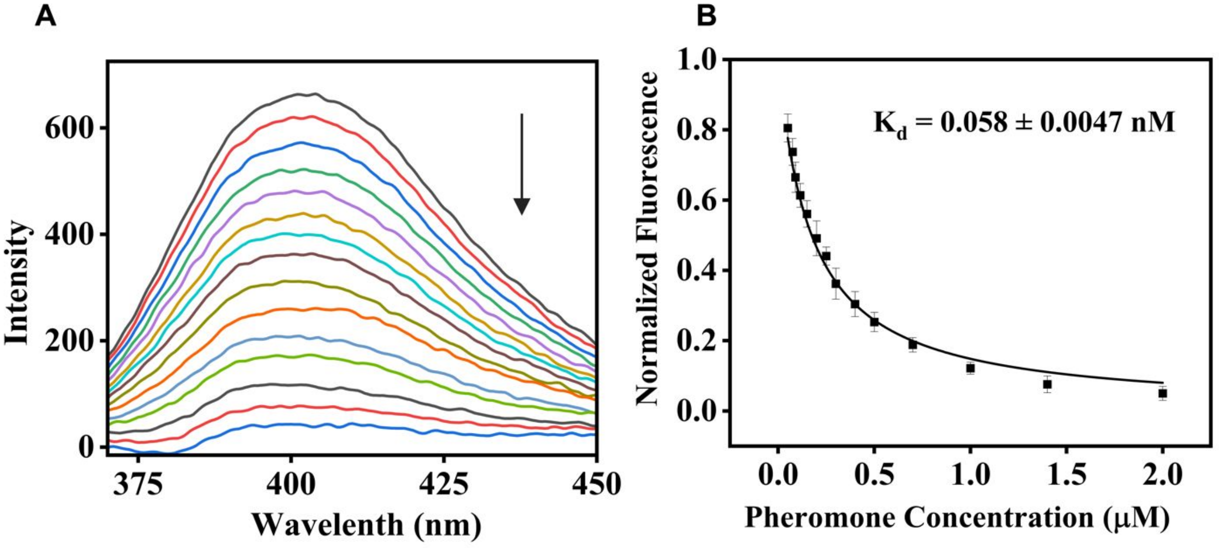

Figure S16: Competitive displacement of NPN in ApolPBP1–NPN complex by the pheromone, (6*E*,11*Z*)-hexadeca-6,11-dienyl-1-acetate: (A) Fluorescence emission spectra of the ApolPBP1–NPN complex recorded upon titration with increasing concentrations of the pheromone. (B) Plot of normalized fluorescence intensity as a function of pheromone concentration.

Ligplot diagram for ApolPBP1-ligand complex

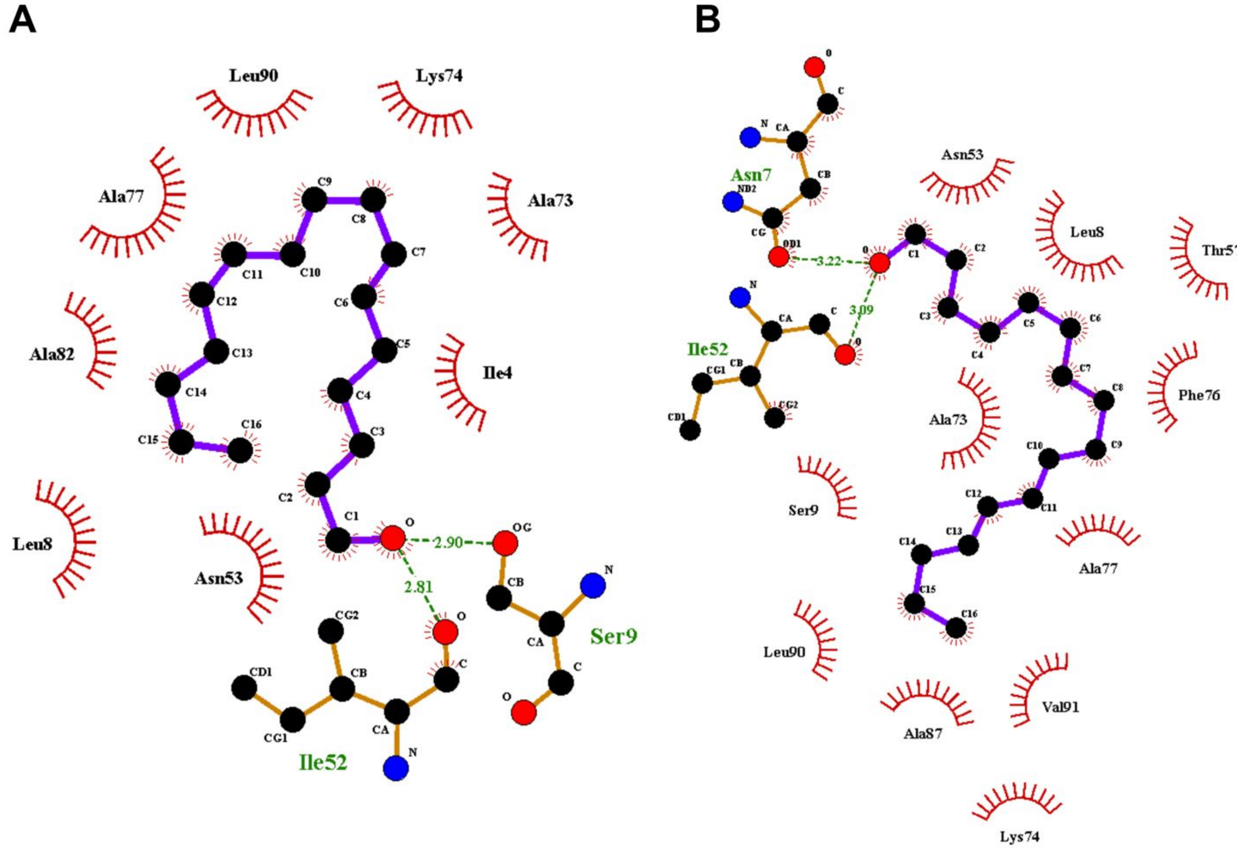

Figure S17: LigPlot diagram showing the interactions between ApolPBP1 and the ligands. (A) Interactions between ApolPBP1 and the pheromone metabolite, (6*E*,11*Z*)-hexadeca-6,11-dien-1-ol. (B) Interactions between ApolPBP1 and bombykol, (10*E*,12*Z*)-hexadeca-10,12-dien-1-ol.

Docking predicted binding interactions and energies

Table S1: CB-Dock Predicted Binding Interactions and Energies

Ligand Type	Ligand Name	Key Residues Involved	Binding Energy (kcal/mol)
Natural pheromone (*Antheraea polyphemus*)	(6*E*,11*Z*)-hexadeca-6,11-dien-1-yl acetate)	Ile4, Asn7, Leu8, Ser9, Ile52, Asn53, Thr57, Ala73, Phe76, Ala77	**-5.7**
Pheromone analog	(6*E*,11*Z*)-heptadeca-6,11-dien-1-yl acetate)	Ile4, Asn7, Leu8, Ile52, Asn53, Ala56, Thr57, Ala73, Lys74, Phe76, Ala77, Ala87, Leu90, Ile94	**-5.4**
Pheromone metabolite	(6*E*,11*Z*)-hexadeca-6,11-dien-1-ol	Ile4, Leu8, Ser9, Ile52, Asn53, Ala73, Lys74, Ala77, Ala82, Leu90	**-4.9**
Bombykol (*Bombyx mori* pheromone)	(10*E*,12*Z*)-hexadeca-10,12-dien-1-ol	Asn7, Leu8, Ser9, Ile52, Asn53, Thr57, Ala73, Lys74, Phe76, Ala77, Ala87, Leu90, Val91	**-5.3**
